# Cancer therapies activate RIG-I-like receptor pathway through endogenous non-coding RNAs

**DOI:** 10.18632/oncotarget.8420

**Published:** 2016-03-28

**Authors:** Diana Rose E. Ranoa, Akash D. Parekh, Sean P. Pitroda, Xiaona Huang, Thomas Darga, Anthony C. Wong, Lei Huang, Jorge Andrade, Jonathan P. Staley, Takashi Satoh, Shizuo Akira, Ralph R. Weichselbaum, Nikolai N. Khodarev

**Affiliations:** ^1^ Department of Radiation and Cellular Oncology and Ludwig Center for Metastasis Research, The University of Chicago, Chicago, IL 60637, USA; ^2^ Center for Research Informatics, Biological Sciences Division, The University of Chicago, Chicago, IL 60637, USA; ^3^ Department of Molecular Genetics and Cell Biology, The University of Chicago, Chicago, IL 60637, USA; ^4^ Laboratory of Host Defense, WPI Immunology Frontier Research Center, Osaka University, Suita, Osaka 565-0871, Japan

**Keywords:** ionizing radiation, DNA damage, small non-coding RNAs, Type I interferon, RIG-I-like receptor (RLR)

## Abstract

Emerging evidence indicates that ionizing radiation (IR) and chemotherapy activate Type I interferon (IFN) signaling in tumor and host cells. However, the mechanism of induction is poorly understood. We identified a novel radioprotective role for the DEXH box RNA helicase LGP2 (*DHX58*) through its suppression of IR-induced cytotoxic IFN-beta [1]. LGP2 inhibits activation of the RIG-I-like receptor (RLR) pathway upon binding of viral RNA to the cytoplasmic sensors RIG-I (*DDX58*) and MDA5 (*IFIH1*) and subsequent IFN signaling via the mitochondrial adaptor protein MAVS (*IPS1*). Here we show that MAVS is necessary for IFN-beta induction and interferon-stimulated gene expression in the response to IR. Suppression of MAVS conferred radioresistance in normal and cancer cells. Germline deletion of RIG-I, but not MDA5, protected mice from death following total body irradiation, while deletion of LGP2 accelerated the death of irradiated animals. In human tumors depletion of RIG-I conferred resistance to IR and different classes of chemotherapy drugs. Mechanistically, IR stimulated the binding of cytoplasmic RIG-I with small endogenous non-coding RNAs (sncRNAs), which triggered IFN-beta activity. We demonstrate that the small nuclear RNAs U1 and U2 translocate to the cytoplasm after IR treatment, thus stimulating the formation of RIG-I: RNA complexes and initiating downstream signaling events. Taken together, these findings suggest that the physiologic responses to radio-/chemo-therapy converge on an antiviral program in recruitment of the RLR pathway by a sncRNA-dependent activation of RIG-I which commences cytotoxic IFN signaling. Importantly, activation of interferon genes by radiation or chemotherapy is associated with a favorable outcome in patients undergoing treatment for cancer. To our knowledge, this is the first demonstration of a cell-intrinsic response to clinically relevant genotoxic treatments mediated by an RNA-dependent mechanism.

## INTRODUCTION

Accumulating data indicate a link between ionizing radiation (IR) and interferon (IFN) signaling. IFN signaling activates multiple interferon-stimulated genes (ISGs) and leads to growth arrest and cell death in exposed cell populations [2–5]. It has been demonstrated that IR-induced tumor-derived type I IFN production is important for improved tumor responses [6, 7], suggesting that Type I IFN is an essential part of IR-delivered tumor cytotoxicity and/or activation of the immune system [3, 8, 9]. However, molecular mechanisms governing tumor cell-intrinsic IR-mediated IFN activation are largely unknown.

Recently we identified DEXH box RNA helicase LGP2 (*DHX58*) as a negative regulator of IR-induced cytotoxic IFN-beta production contributing to cell-autonomous radioprotective effects in cancer cells [1]. LGP2 is a cytoplasmic RIG-I-like receptor (RLR) which suppresses IFN signaling in the response to viral double-stranded RNA [10]. RLRs are members of pattern recognition receptors (PRRs) which mediate the induction of IFN signaling in the response to pathogens due to abnormal accumulation of ribonucleic acids in the cytoplasm or extracellular space [11]. RLRs are part of innate immune responses that evolved in eukaryotic cells as protective mechanisms against pathogens following recognition of foreign macromolecules. Specific for these foreign organisms (see [11–14] for reviews). Identification of LGP2 as a regulatory protein involved in the IR response implicated RNA recognition systems in the damage response to IR, which has typically been associated with DNA damage recognition signaling.

**Figure 1:**
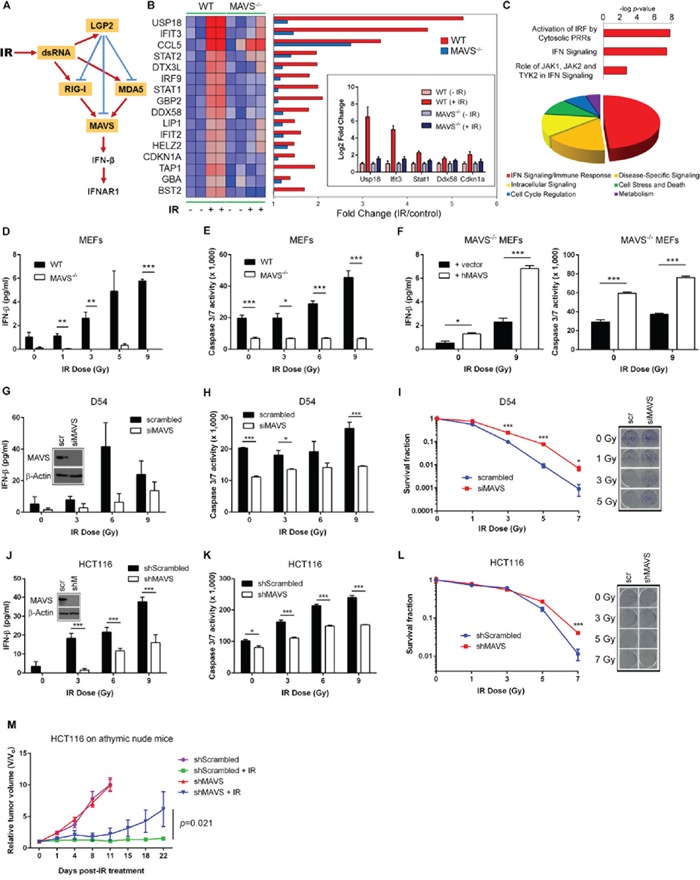
MAVS is necessary for ionizing radiation-induced Type I interferon signaling **A.** Proposed mechanism of MAVS-dependent activation of Type I IFN signaling in the cellular response to IR. **B.** Transcriptional profiling of C57BL/6 wild-type (WT) and MAVS^−/−^ primary MEFs demonstrating MAVS-dependent expression of Type I IFN-stimulated genes (ISGs) 48 hours following exposure to IR (6 Gy). Heatmap displays differences in gene expression values between WT and MAVS^−/−^ MEFs; red indicates high expression and blue low expression. Inset shows qRT-PCR validation of *Usp18*, *Ifit3*, *Stat1*, *Ddx58*, and *Cdkn1a* gene expression values in WT and MAVS^−/−^ MEFs after IR treatment. **C.** Top-ranked cellular pathways (top) and functions (bottom) (Ingenuity Pathway Analysis) activated by IR in WT MEFs. Pie-chart displays the relative abundance of each functional category among all significant functions (*P* < 0.05). IRF – interferon regulatory factor; PRR – pattern recognition receptor; JAK – Janus kinase; TYK – tyrosine kinase. IFN-beta protein secretion **D.** and caspase 3/7 activity **E.** in WT and MAVS^−/−^ MEFs 48 hours following exposure to increasing doses of IR. **F.** IFN-beta protein secretion and caspase 3/7 activity 48 hours following IR exposure of MAVS^−/−^ MEFs reconstituted by transient transfection of a full-length human MAVS construct (hMAVS) or an empty vector control (vector). IR-induced IFN-beta **G.**, caspase 3/7 activity **H.** and clonogenic survival **I.** following siRNA-mediated suppression of MAVS (siMAVS) in human D54 glioblastoma cells. Scr - scrambled siRNA control. IR-induced IFN-beta **J.**, caspase 3/7 activity **K.** and clonogenic survival **L.** following stable shRNA-mediated suppression of MAVS (shMAVS) in human HCT116 colorectal carcinoma cells. Depletion of MAVS increased D_o_ values (dose required to reduce the fraction of surviving cells to 37%) from 1.01 ± 0.02 Gy to 1.43 ± 0.1 Gy (*P*=0.0025) in D54 and from 1.67 ± 0.22 Gy to 2.36 ± 0.09 Gy (*P*=0.0074) in HCT116 cells. Western blot analysis and representative scanned images of culture dishes after MAVS depletion and subsequent IR treatment are shown in the insets for (G), (I), (J), and (L). shM – shMAVS. Data are representative of three independent experiments. **M.** Relative tumor growth of shMAVS HCT116 tumor xenografts in athymic nude mice treated with IR (5 Gy x 6 daily fractions). Data are representative of two experiments, each with n = 5 mice per group. *P* values were determined using unpaired Student's *t*-test. Error bars are SEM. *P < 0.05, **P < 0.01, ***P < 0.005.

RLRs are represented by 3 major primary RNA sensors (RIG-I, MDA5 and LGP2) and one common adapter protein MAVS (Mitochondrial anti-viral signaling protein-see Figure 1A). Upon recognition of specific double-stranded RNA molecules, RIG-I and MDA5 expose their CARD domains to allow interaction and subsequent activation of MAVS (see [15-17]). Activated MAVS recruits IRF3 and NFkB and eventually leads to the activation of IFN-beta, through multiple intermediate steps which are still under investigation. LGP2 has context-specific functions, but often acts as the suppressor of RNA-dependent IFN-beta production (see [10, 18] for reviews), consistent with our observations of the LGP2 functions in the response of various types of tumor cells to IR [1].

RIG-I and MDA5 are able to recognize foreign viral RNAs based on their primary and secondary structure, size, structure of 5′ends of RNAs and/or recognition of methylated patterns in the 5′capping structures of RNAs [15, 19, 20]. As well, the concentration of RNAs in the cytoplasmic fraction may be important in activation of these primary RNA sensors [21].

In the current paper we used a combination of genetic, biochemical and bioinformatics approaches to systematically investigate the effects of the each component of RLR pathway on the ability of IR and chemotherapy to kill normal and tumor cells and produce IFN-beta. Our data indicate that the RLR pathway is necessary and sufficient in the ability of IR and chemotherapy to induce a cytotoxic response and IFN-beta production. We also show that the RLR pathway is activated by endogenous small non-coding RNAs that accumulate in the cytoplasm in response to genotoxic stress and bind to RIG-I to activate downstream IFN-beta production. RLR pathway confers tumor responses in *in vivo* xenograft models and is responsible for the lethal gastrointestinal injury after total body irradiation (TBI). Finally, analysis of available databases demonstrated that the RLR pathway is involved in the response to radio/chemotherapy in cervical, breast, bladder and rectal cancers, which supports the design of appropriate biomarkers for clinical applications and search for druggable targets regulating this pathway [9, 22, 23].

## RESULTS

### MAVS is necessary and sufficient for the ability of IR to induce IFN signaling and cell killing

We explored the role for RLR signaling in the response to IR. We hypothesized that following irradiation, endogenous RNA moieties are upregulated in the cytoplasm and thereby recognized by cytoplasmic RNA sensors (Figure 1A). Irradiation (6 Gy) induced the overexpression of 82 genes in C57BL/6 wild-type (WT) primary mouse embryonic fibroblasts (MEFs) at 48 hours following treatment. Sixteen of these genes were identified as type I ISGs (Figure 1B and 1C). Notably, expression of RIG-I (*DDX58*), but not MDA5 (*IFIH1*), was induced by IR. In contrast, MAVS^−/−^ MEFs failed to induce type I ISG expression in irradiated cells (Figure 1B). IR led to a dose-dependent accumulation of IFN-beta in WT MEFs which was absent in MAVS^−/−^ MEFs (Figure 1D). Consistently, Western blot analyses reveal that MAVS^−/−^ MEFs have lower phosphorylated TBK1 and basal IRF3 levels compared to the WT controls in response to increasing doses of IR (Supplementary Figure S1A). WT MEFs also demonstrated an IR dose-dependent activation of caspases 3/7 which was blunted in MAVS^−/−^ MEFs (Figure 1E). The differences in caspase activation paralleled differences in clonogenic survival of WT and MAVS^−/−^ SV40-transformed MEFs (Supplementary Figure S1B). Reconstitution of MAVS restored IFN-beta production and IR-induced caspase activation in MAVS^−/−^ MEFs (Figure 1F). Consistent with these findings, IR induced a cytotoxic IFN-beta response in human D54 glioblastoma (Figure 1G-1I) and HCT116 colorectal carcinoma cell lines (Figure 1J-1L) which was suppressed by MAVS depletion. Interestingly, basal production and IR-induced levels of secreted IFN-beta were higher in tumor cells as compared with primary fibroblasts. MAVS knockdown in WiDr human colon adenocarcinoma cells also conferred radioresistance (Supplementary Figure S1C). We then investigated the response to IR of the corresponding tumors established as hind limb xenografts in athymic nude mice. As shown in Figure 1M, depletion of MAVS led to a significant tumor regrowth following IR with no apparent effect on untreated tumors.

Type I IFN receptor signaling was necessary for the cell death following IR exposure as evidenced by suppression of IR-induced apoptosis after administration of neutralizing anti-IFNAR1 monoclonal antibody (Supplementary Figure S1D). Taken together, these data demonstrated that MAVS-dependent signaling confers IR-mediated cytotoxicity through IFN-beta production.

### RIG-I is the critical RNA sensor responsible for IR-induced and chemotherapy-induced cell killing

**Figure 2:**
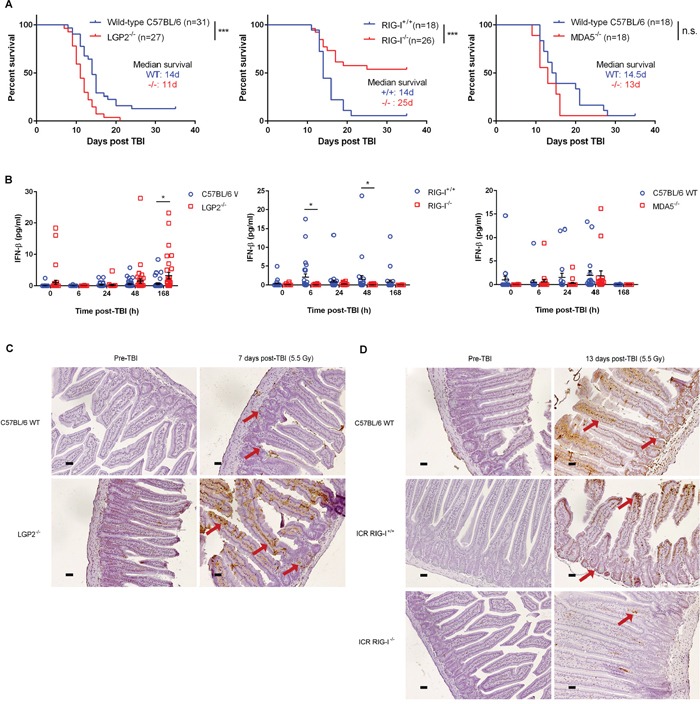
RLR pathway mediates radiation-induced gastrointestinal death following total body irradiation **A.** Overall survival following total body irradiation (TBI, 5.5 Gy) of age-matched (9-12 weeks) wild-type (C57BL/6 or ICR background) and germline deleted LGP2^−/−^ (left), RIG-I^−/−^ (middle), and MDA5^−/−^ (right) mice. Differences in survival were assessed using log-rank tests. *P < 0.05, **P < 0.01, n.s. – not significant. **B.** IFN-beta quantification in mouse serum at specified time-points following exposure to TBI (5.5 Gy). Horizontal bar denotes mean value. Error bars are SEM. **C.** Small intestinal TUNEL staining of C57BL/6 wild-type (WT) and LGP2^−/−^ mice prior to and 7 days following total body irradiation at 5.5 Gy. Small intestinal cross-sections from LGP2^−/−^ mice exhibited greater intestinal crypt destruction (denoted by red arrows) as well as increased apoptosis (brown staining) in the crypt cells and the enterocytes lining the microvilli as compared to wild-type mice. **D.** Small intestinal TUNEL staining of C57BL/6 wild-type (WT), ICR RIG-I^+/+^ WT and ICR RIG-I^−/−^ mice prior to and 13 days following total body irradiation at 5.5 Gy. Small intestinal cross-sections from RIG-I^−/−^ mice showed minimal apoptotic staining in the enterocytes lining the microvilli as compared to wild-type mice. All images are representative of three replicates per condition. Magnification, 20x; scale bars, 0.11 μm.

RNA sensing via MAVS-dependent signaling is mediated by three RNA sensors – LGP2, RIG-I and MDA5. RIG-I and MDA5 promote MAVS activation, while LGP2 is thought to regulate RIG-I and MDA5 in cell- and viral-specific contexts [24–26]. We tested whether LGP2, RIG-I, and MDA5 contribute to the total body irradiation (TBI; 5.5 Gy) response. We found that LGP2 conferred radioprotection, while RIG-I mediated radiosensitivity (Figure 2A). LGP2 expression inversely correlated with IFN-beta secretion, whereas RIG-I promoted IFN-beta production in the response to TBI (Figure 2B). LGP2^−/−^ mice demonstrated elevated levels of apoptosis in intestinal crypt cells and epithelial cells comprising the microvilli and lamina propria as compared to wild-type animals (Figure 2C), which is consistent with death due to radiation-induced gastrointestinal injury [27, 28]. In contrast, RIG-I^−/−^ mice showed minimal IR-induced intestinal apoptosis and exhibited higher survival rates compared to the RIG-I^+/+^ controls (Figure 2D). On the other hand, MDA5 exerted no measurable effect on radiosensitivity or IFN-beta production (Figure 2A).

At the cellular level, LGP2^−/−^ MEFs exhibited increased IFN-beta production, caspase 3/7 activation, and decreased clonogenic survival after IR exposure (Supplementary Figure S2). These data supported the notion that LGP2 suppresses IR-induced RIG-I-dependent IFN-beta signaling [1]. The data indicated that RLR-dependent Type I IFN production is an important component of the lethal effects of IR, which may contribute to the GI death, induced by TBI.

**Figure 3:**
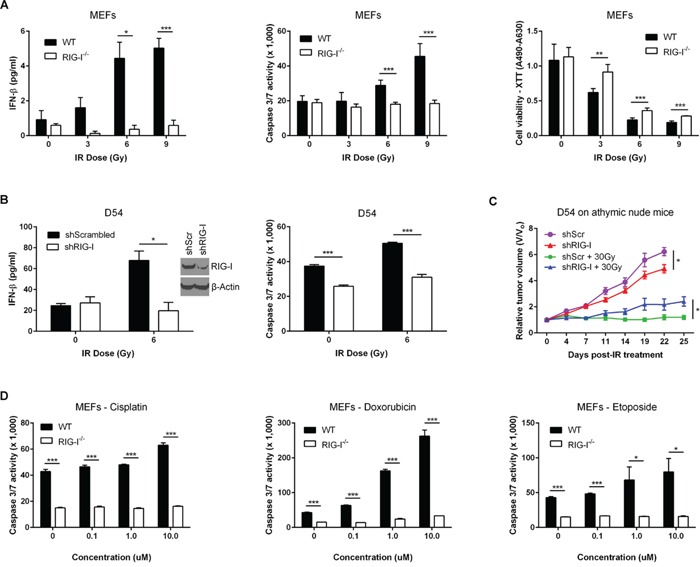
RIG-I orchestrates the MAVS-dependent Type I interferon response to ionizing radiation **A.** Quantification of IR-induced IFN-beta secretion (left), caspase 3/7 activation (middle), and cell viability using XTT assay (right) in ICR RIG-I^+/+^ (WT) and RIG-I^−/−^ MEFs 48 hours after IR exposure. **B.** IFN-beta protein secretion (left) and caspase 3/7 activation (right) 48 hours post-IR treatment following shRNA-mediated suppression of RIG-I (shRIG-I) in D54 cells. shScrambled – scrambled shRNA control. **C.** Relative tumor growth of shRIG-I D54 tumor xenografts in athymic nude mice treated with IR (5 Gy x 6 daily fractions). shScr – scrambled shRNA control. Data are representative of three experiments, each with n = 5 mice per group. **D.** Caspase 3/7 activity of RIG-I^−/−^ and WT MEFs in response to increasing doses of cisplatin (left), doxorubicin (middle) and etoposide (right). Data are representative of three independent experiments. *P* values were determined using unpaired Student's *t*-test. Error bars are SEM. *P < 0.05, **P < 0.01, ***P < 0.005.

We further examined the relative contributions of RIG-I and MDA5 in IFN-beta induction after exposure to IR. Ectopic expression of MAVS or RIG-I activated the IFN-beta promoter in an IR-dependent manner (Supplementary Figure S3A and S3B). In contrast, overexpression of MDA5 led to a modest activation of IFN-beta at the basal level, but not by IR (Supplementary Figure S3C). We therefore focused on the role of RIG-I in IR-induced cytotoxicity. We found that irradiated RIG-I^−/−^ MEFs were deficient in both the IFN-beta response and caspase 3/7 activity, and demonstrated increased survival as compared to wild-type MEFs (Figure 3A). Reconstitution of RIG-I^−/−^ MEFs by full-length RIG-I restored radiosensitivity (Supplementary Figure S3D). Similarly, D54 and HCT116 tumor cells depleted of RIG-I exhibited suppression of IFN-beta secretion and caspase 3/7 responses to IR as well as radioresistance in clonogenic assays (Figure 3B and Supplementary Figure S4a-S4c). To test the effects of tumor cell-derived IFN on *in vivo* growth and radioresistance, we established D54 human tumor xenografts with stable suppression of RIG-I in athymic nude mice (Figure 3C). In the absence of radiation, depletion of RIG-I reduced tumor growth rate as compared to control cells. In contrast, tumor regrowth was greater in RIG-I knockdown tumors after IR treatment. Collectively, these data supported a critical role for RIG-I in mediating the RLR response of normal and tumor cells to IR.

Recently it was demonstrated that treatment of fibrosarcomas with anthracyclines, such as doxorubicin, led to a cell-autonomous induction of ISGs via Toll-like receptor 3 but not the cytosolic sensor MDA5 [29]. We used three different classes of chemotherapy drugs (platinum – cisplatin, anthracycline – doxorubicin (Adriamycin) and topoisomerase II inhibitor – etoposide) to test the effects of RIG-I on the response to these drugs. Our results show that the absence or depletion of RIG-I reduced caspase 3/7 activity in the response to treatment when compared to control cells (Figure 3D and Supplementary Figure S4D). Taken together, these data suggest that RIG-I is important for cell-intrinsic IFN production in the response to multiple classes of genotoxic anticancer therapies.

### RIG-I is activated by IR-induced endogenous double-stranded RNAs

**Figure 4:**
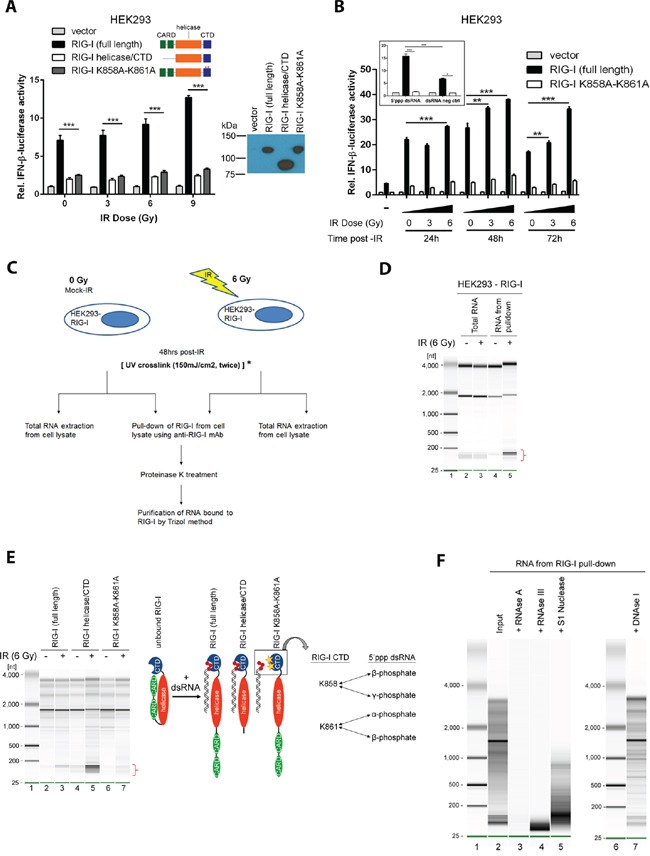
IR induces RIG-I binding to endogenous double-stranded RNAs **A.** HEK293 reporter cells were irradiated after transfection with either an empty vector, a full length human RIG-I, a RIG-I lacking CARD domains (RIG-I helicase/CTD), or a RIG-I harboring K858A and K861A mutations in the C-terminal domain (RIG-I K858A-K861A), in addition to an IFN-beta promoter-driven luciferase construct. A Renilla reporter construct served as a transfection control. Data are presented as mean fold-change relative to the non-irradiated empty vector control. **B.** Donor HEK293 cells were either unirradiated or treated with IR (3 or 6 Gy). Total RNA was purified and transferred to independent batches of HEK293 reporter cells transfected by RIG-I constructs as described in (A). A synthetic double-stranded RNA construct comprised of 5′-triphosphorylated dsRNA and an unphosphorylated counterpart served as positive and negative controls, respectively (inset). **C.** Experimental design for isolation and purification of RNA bound to RIG-I after exposure to IR. *To validate RNA sequencing data by qPCR experiments, UV crosslinking was performed prior to cell lysis and immunoprecipitation of RIG-I. See methods for further details. **D.** Purified RNA from total cellular extracts (Lanes 2 and 3) and complexes with RIG-I (Lanes 4 and 5). Lane 1 is the marker. Data are representative of at least 3 independent experiments. **E.** HEK293 cells over-expressing the HA-tagged full length RIG-I (Lanes 2 and 3), the RIG-I helicase-CTD mutant (Lanes 4 and 5) and the RIG-I K858A-K861A CTD mutant (Lanes 6 and 7) were either un-irradiated or exposed to IR (6 Gy), lysed and incubated with anti-HA monoclonal antibody to pulldown the respective WT and mutant RIG-I proteins. RIG-I diagrams illustrate the mechanism of RIG-I activation (adapted from [57]). In the inactive/unbound conformation, the CARD domain of RIG-I is folded to block the helicase domain from RNA binding RNA, but allows the CTD to search for its ligand. Upon binding of the blunt end of a dsRNA molecule to the CTD, the CARD domain opens to allow the helicase domain to bind the remaining dsRNA molecule. Absence of the CARD domain in the helicase/CTD mutant enables higher affinity binding to dsRNA ligands as compared to the full length RIG-I. The lysine residues at amino acid positions 858 and 861 have previously demonstrated importance in latching onto the 5′-triphosphorylated end of viral dsRNA ligands. **F.** RNA bound to RIG-I after exposure to IR (6 Gy) was treated with: RNase A (lane 3), dsRNA-specific RNase III (lane 4), single-strand specific nuclease S1 (lane 5) and DNase I (lane 7). Lane 2 shows the input and lanes 1 and 6 display markers.

RIG-I is an RNA binding protein with two caspase recruitment domains (CARD) responsible for MAVS activation, an RNA helicase domain, and a C-terminal domain which determines the primary binding of 5′-phosphorylated dsRNA [30]. Expression of the full-length RIG-I protein in HEK293 reporter cells led to an IR dose-dependent activation of the IFN-beta promoter (Figure 4A). In contrast, deletion of both CARDs or mutations of C-terminal amino acids at positions K858 and K861, which are important for efficient RNA binding, abrogated IR-mediated IFN-beta expression [31, 32]. These findings supported a role for the RNA binding function of RIG-I in transduction of IR-dependent IFN signaling. We tested the hypothesis that IR induces the expression of RIG-I-activating RNAs. HEK293 IFN-beta luciferase reporter cells transfected with a full-length RIG-I, a K858A-K861A RNA binding deficient mutant, or an empty vector were stimulated with total RNA purified from control or irradiated donor HEK293 cells (Figure 4B). HEK293 cells expressing full-length RIG-I, but not the RNA binding deficient K858A-K861A, demonstrated IFN-beta induction in a dose- and time-dependent manner (Figure 4B). We therefore concluded that IR leads to the appearance of RNA species, able to activate RIG-I through its RNA binding pocket.

We further immunoprecipitated RNA bound to ectopically expressed RIG-I following IR (see scheme of the experiments in Figure 4C). Non-irradiated and isotype control samples contained no detectable RNA, while, in contrast, we detected RNA in RIG-I complexes following IR (Figure 4D). IR led to an enrichment of small RNA molecules (~180 nucleotides) in RIG-I complexes (Figure 4D lane 5 and Figure 4E lane 3). As compared to full-length RIG-I, CARD deletion increased RNA binding, consistent with recent findings [33] (Figure 4E lane 5). In contrast, K858A-K861A RIG-I mutations diminished RNA binding (Figure 4E lane 7). RIG-I-bound material was RNase A-sensitive, DNase I-resistant, partially resistant to single-stranded specific nuclease S1 but sensitive to double-stranded specific nuclease RNase III (Figure 4F). These results indicated that RIG-I binds RNA molecules enriched with double-stranded regions, which is consistent with the known substrate specificity of the RIG-I protein [34]. Taken together, these findings suggested IR-induced activation of IFN signaling occurs through binding of endogenous RNA molecules which contain double-stranded regions with the C-terminal K858-K861 pocket of the RIG-I protein (see inset of Figure 4E for the illustration).

### Nuclear-cytoplasmic distribution of small non-coding RNAs leads to RIG-I – mediated IFN-beta response

**Figure 5:**
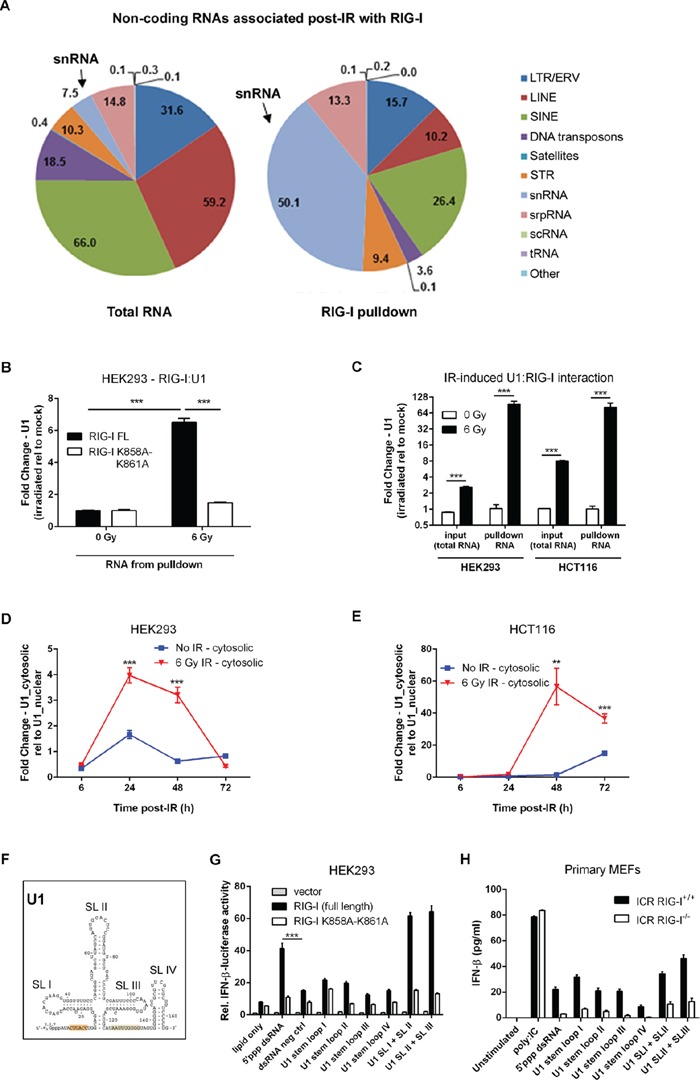
RIG-I binds U1 snRNA accumulated in the cytoplasm to mediate radiation-induced IFN-beta response **A.** RIG-I binds diverse non-coding RNA molecules, majority of which are snRNAs. Graphic representation indicating the distribution of non-coding and repetitive RNA molecules bound to RIG-I following exposure to IR as compared to total irradiated cellular RNA. Transcripts were mapped to reference genomes using RepeatMasker. See Methods for further details. **B.** qRT-PCR quantification of U1 RNA from purified RNA bound to ectopically expressing WT and K858A-K861A mutant RIG-I HEK293 cells exposed to IR (6 Gy) or left untreated. Cells were UV crosslinked at 150mJ/cm^2^ 48 hours post-IR treatment prior to cell lysis. U1 RNA levels were normalized to the geometric average of 3 housekeeping genes (18S rDNA, GAPDH, and β-actin). Fold change was determined relative to un-irradiated controls. **C.** U1 RNA levels quantified by qRT-PCR from total cellular and RIG-I pulldowns in RIG-I overexpressing HEK293 and HCT116 cells. U1 RNA levels were normalized to the geometric average of 3 housekeeping genes (18S rDNA, GAPDH, and β-actin). Fold change was determined relative to un-irradiated controls. Time course of cytosolic accumulation of U1 RNA measured by qRT-PCR from purified total cellular RNA following cellular fractionation of nuclear/mitochondrial and cytoplasmic fractions of HEK293 **D.** and HCT116 cells **E.** exposed to IR (6 Gy) or left untreated. **F.** The structure of the U1 snRNA illustrating the four stem loop (SL) regions. **G.** Relative IFN-beta luciferase reporter activity of HEK293 cells following a 24 hour stimulation with synthetic oligonucleotides corresponding to U1 RNA stem loop (SL) regions I to IV or a combination of SL I + II and SL II + III. **H.** IFN-b levels in culture supernatant from ICR RIG-I^+/+^ and RIG-I^−/−^ primary MEFs 24 hours post-stimulation with the same set of synthetic U1 oligonucleotides used in G. The amount of U1 synthetic oligonucleotides used in all stimulation experiments was 1μg. *P* values were determined using unpaired Student's *t*-test. Error bars are SEM. ***P < 0.005.

Previous reports indicate that genotoxic stress activates the transcription of repetitive and non-coding RNAs [35–37]. We used an RNA sequencing approach to preliminarily characterize RNAs bound to RIG-I post-IR. The most striking result of these experiments was an enrichment of RIG-I by the small nuclear RNAs U1 and U2 following IR (Figure 5A and Supplementary Table 1). To validate these pilot data, we used a combination of covalent UV-cross-linking with quantitative real-time PCR (CLIP-PCR). We found a 6-fold enrichment of U1 and U2 snRNA in purified RIG-I complexes from irradiated HEK293 cells as compared to non-irradiated controls (Figure 5B and Supplementary Figure S5A). We did not detect increased levels of either U1 or U2 in HEK293 cells overexpressing the K858A-K861A RNA binding deficient mutant RIG-I. We should note that this is the most stringent negative control for these types of experiments, clearly demonstrating that IR induces specific binding of U1 and U2 to RIG-I. Importantly, pull-down of RIG-I: RNA complexes from HCT116 cells overexpressing RIG-I also demonstrated a significant enrichment by U1 and U2 in irradiated samples indicating a similar mechanism of RIG-I activation in tumor cells (Figure 5C and Supplementary Figure S5B). Given that small nuclear RNAs predominantly reside in the nucleus, we hypothesized that following IR, U1 and U2 snRNAs translocate to the cytoplasm which permits interaction with RIG-I. Indeed, we observed a cytoplasmic redistribution of U1 and U2 RNAs following IR exposure in both HEK293 and HCT116 cells (Figure 5D-5E and Supplementary Figure S5C-S5D). In HEK293 cells, there was a 4-fold increase in the nuclear/cytoplasmic ratio of U1 RNA at 24 hours post-IR as compared to untreated cells (Figure 5D). Similar dynamics were observed in HCT116 cells (Figure 5E). Likewise, we observed cytoplasmic re-distribution of the U2 snRNA in both HEK293 and HCT116 cell lines starting at 24 hours post-IR (Supplementary Figure S5C-S5D). Interestingly, higher cytoplasmic/nuclear ratios of U1 RNA levels in HCT116 cells as compared to HEK293 cells correlated with previous observations showing elevated levels of IFN-beta production in tumor cells relative to normal cells (Figure 1D-1F). Importantly, IR also induced the cytoplasmic accumulation of RIG-I protein both in primary MEFs and in at least two different tumor cell lines (Supplementary Figure S6). Thus far, our data suggest that activation of RLR signaling by genotoxic stress is associated with nuclear to cytoplasmic redistribution of U1 (and U2) and the radio-inducibility of RIG-I.

To further confirm that RIG-I recognition of U1 induces IFN-beta signaling, we used *in vitro* transcribed (IVT) full length U1 RNA as an agonist in our HEK293 dual luciferase reporter system. We demonstrated that U1 RNA has potent IFN-beta stimulatory activity in RIG-I overexpressing cells and is able to activate endogenous RIG-I in HEK293 cells (Supplementary Figure S7A). Digestion of U1 RNA by RNAse III markedly diminished RIG-I-dependent IFN-beta activation, indicating the importance of double-stranded regions of this molecule for induction of IFN response. Furthermore, treatment of U1 with calf intestinal alkaline phosphatase (CIAP) to remove the phosphate group at the 5′ end reduced IFN-beta reporter activity by two-fold (Supplementary Figure S7B). To assess this response in further detail, we chemically synthesized stem loop (SL) regions of U1 (Figure 5F). We found that double-stranded regions of U1 (SL I + II or SL II + III) are potent inducers of IFN-beta response (Figure 5G and 5H). Interestingly, the same sequences of U1 have been reported to induce cytokine production in keratinocytes following exposure to ultraviolet radiation in a Toll-like receptor (TLR) 3-dependent manner [38]. These data support the notion that U1 is a potential endogenous activating ligand for RIG-I. Taken together, these data suggest that cell-intrinsic cytosolic accumulation of RIG-I: RNA complexes in irradiated cells activates MAVS-dependent IFN-signaling.

### RIG-I signaling confers the response to DNA-damaging therapy

**Figure 6:**
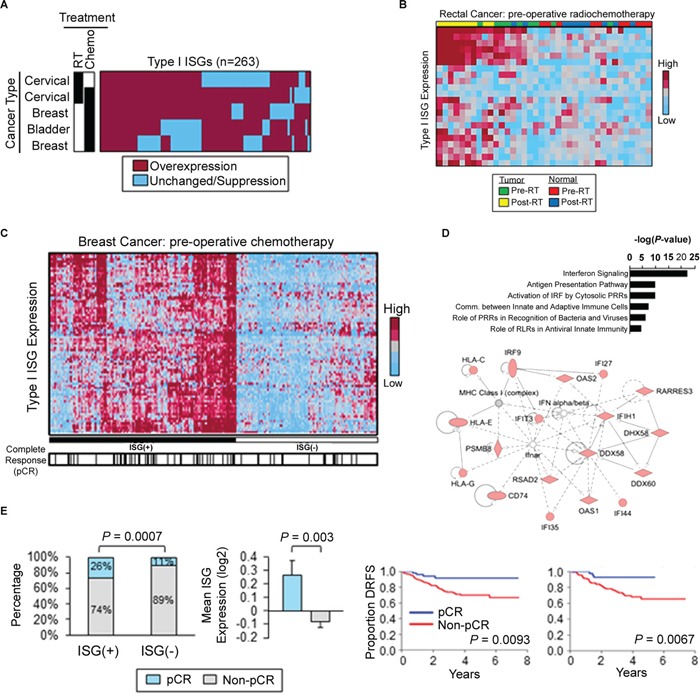
Radiation and chemotherapy activate Type I interferon-stimulated genes in cancer patients **A.** Heatmap displaying the commonality of Type I ISG induction in human cervical, breast, and bladder cancers following genotoxic treatment. Black boxes denote treatment. Gene expression values were obtained from microarray analysis of matched pre- and post-treatment tumor biopsies. Overexpression defined as fold-change > 1 in post-treatment biopsies as compared to matched pre-treatment biopsies. **B.** Type I ISG expression in pre- and post-chemoradiation specimens of human rectal cancer and matched normal tissue. **C.** Type I ISGs (n=81) distinguish breast cancer patients (GSE25055, n=310). ISG(+) defined by overexpression of type I ISGs (left). Black hash marks denote complete pathologic response (pCR) to pre-operative doxorubicin-based chemotherapy. **D.** Canonical pathways (top) and top-ranked gene network (bottom) from Ingenuity Pathway Analysis of Type I ISGs identified in (C). **E.** (Left) Frequency of pCR in ISG(+) and ISG(-) breast cancers treated with pre-operative doxorubicin-based chemotherapy. *P* value was determined by using Fisher's exact test. (Middle) Mean ISG expression (81 genes) in breast cancers which achieved a pCR to pre-operative chemotherapy vs. tumors with residual disease (non-pCR). *P* value was determined by using unpaired Student's *t*-test. Error bars are SEM. (Right) Kaplan-Meier estimates of distant relapse-free survival (DRFS) in breast cancer patients with a pCR vs. non-pCR. Left: GSE25055 (n=310); right: GSE25065 (n=198). *P* values were determined by using log-rank tests.

Based on our experimental data, we hypothesized that DNA damaging therapies induce Type I ISG expression in cancer patients. Of 371 Type I ISGs [39], 263 (71%) were induced in cervical, breast, and bladder cancers in the responses to genotoxic treatments (Figure 6A). Tumors exhibited elevated ISG expression pre- and post-treatment in patients treated with radiotherapy and chemotherapy as compared to corresponding normal tissue (Figure 6B). These findings are consistent with previous data demonstrating elevated levels of IFN signaling in tumor cells (Figure 1G and 1J). We identified an 81-gene subset of treatment-responsive ISGs that predicted a complete pathologic response (pCR) to pre-operative doxorubicin-based chemotherapy in a data set of 310 breast cancer patients (Figure 6C). These findings were validated in an independent breast cancer data set of 278 patients (Extended Data Figure S8A). Functional analysis of these ISGs highlighted functions mediating activation of IFN by cytosolic pattern recognition receptors and communication between innate and adaptive immune cells (Figure 6D). Quantitatively, ISG(+) tumors were approximately 2.0-fold more likely to achieve a pCR as compared to ISG(-) tumors (Figure 6E and Extended Data Figure S8B). Importantly, the lack of pCR following pre-operative chemotherapy was associated with increased rates of distant relapse in two independent data sets totaling 588 patients (Figure 6E). These findings demonstrate that DNA damaging therapies induce Type I interferon responses in multiple human tumors and support a link between Type I ISG expression and treatment efficacy for breast cancer patients.

## DISCUSSION

Recently, a growing body of evidence indicate a link between radio/chemotherapy of different types of tumors and Type I IFN signaling [2-7, 21, 29], recently reviewed in [9, 40–43]. Type I IFNs induced by genotoxic stress in tumor cells may significantly modulate the response of tumors to radio/chemotherapy. Through autocrine signaling they can sensitize tumor cells to genotoxic treatments and modulate the mode of the cell death induced by IR [1, 3, 9]. Through paracrine signaling IFNs are responsible for recruitment of immune cells to the tumor microenvironment [6, 7] thereby modulating immune response to anti-tumor therapy. Yet, the molecular mechanisms of this link remained unclear. Our previous data using an siRNA screen of Interferon-Stimulated Genes (ISGs) implicated LGP2 (*DHX58*), a member of the RLR pathway and suppressor of RIG-I/MDA5 signaling, as a key negative regulator of IR-induced IFN responses and thereby acts as a powerful radioprotector in multiple types of cancer cells and tumors [1]. Data presented in the current report indeed demonstrate that the LGP2/RIG-I/MAVS pathway, traditionally associated with recognition of viral RNAs, is necessary and sufficient for radio/chemotherapy to induce IFN signaling. We demonstrated that after treatment by IR/chemotherapy this signaling pathway is induced by small endogenous non-coding RNAs enriched with double-stranded structures, which bind to the cytoplasmic RNA sensor RIG-I. MDA5 seems to be redundant in the context of IR signaling (see Figures 2 and Supplementary Figure S3) and further investigations are necessary to evaluate its role in the response of tumor cells to genotoxic therapies. The relevance of these findings is confirmed by data that transgenic animals deficient in RIG-I are more radioresistant while animals depleted of the suppressor of RLR pathway - LGP2 - are more radiosensitive (see Figure 2). The role of LGP2/RIG-I/MAVS pathway in the IR-induced gastrointestinal injury (GI) is consistent with previous observations of TLR2/3/4 functions in the GI [44] and can provide new targets for intestinal radioprotection. Furthermore, tumors with suppressed MAVS and RIG-I demonstrated clear radioresistance, while clinical data indicate that patients with proficient RIG-I/MAVS pathway are responsive to radio/chemotherapy (Figures 1M, 3C and 8). Taken together, these findings demonstrate that the RLR pathway is an essential component of the tumor response to IR and drugs implicated in the anti-tumor therapy. These data pose intriguing questions about the origin of the dsRNA species as well as their role in mediating cytotoxic insults introduced by traditional DNA-damaging agents.

RNA response to genotoxic stress associated with repetitive and transposable DNA elements in the human and mouse genomes was reported previously. Rubin &Thompson demonstrated that exposure of apoptosis-resistant tumor cells to etoposide, cisplatin and IR led to the up-regulation of repetitive RNA transcripts from Alu1 and SINE elements [36]. Importantly, IR also increased reverse transcriptase (RT) activity associated with endogenous retrotransposons and the capability to transform RNA signals to DNA signals. The cytotoxicity of dsRNA enriched by repetitive AluI elements was further demonstrated in the retinal pigmentum epithelium (RPE) of patients with the age-related macular degeneration (AMD) and was associated with Dicer deficiency [45]. Importantly, toxicity of Alu1 accumulation was conferred by activation of NLRP3 inflammasome and activation of IL18 [37], suggesting involvement of innate immunity pathways in the recognition of endogenous dsRNA and activation of downstream cytokine response. More recently Leonova et al [35] described that DNA demethylation by 5-Aza-dC (inhibitor of DNA-methyltransferase I, DNMT1) leads to the induction of various types of repetitive non-coding dsRNAs, including SINES and microsatellite sequences and is associated with a cytotoxic IFN-beta production and accumulation of ISGs, which overlapped with the IRDS signature previously described by our group [22]. Authors demonstrated that wild-type p53 suppresses induction of these non-coding RNAs thereby acting as a transcriptional repressor of such potentially toxic repetitive dsRNAs. p53-dependence of RNA signaling was also noted in TLR3/TRIF pathway [44]. Recent data from two independent groups confirmed these findings and indicated that DNA demethylation is associated with reactivation of small non-coding RNAs, enriched by endogenous retroviral sequences and associated with activation of TLR3 and/or MDA5/MAVS/IRF7 pathways [46–48]. However, mechanisms of activation of these RNAs and their interaction with specific sensors were not clearly characterized in these publications.

One potential mechanism of the accumulation of toxic dsRNA can occur by the combination of sense- and anti-sense transcription (convergent transcription) of simple trinucleotide repeats (TNRs), usually found in genomic microsatellite sequences. Accumulation of the long (95 TNRs) tracks of such double-stranded transcripts induced apoptosis and led to the death of more than 50% of targeted cells [49, 50]. Convergent transcription can recruit ATR/CHK1/p53 pathway (consistent with data of Leonova et al [35] and Takemura et al [44]) and alter cell cycle progression before induction of cell death [49]. It is unknown whether the LGP2/RIG-I/MAVS pathway is implicated in recognition and signaling from these types of dsRNAs, but considering the high levels of anti-sense transcription in the genome and implication of satellite RNAs in induction of IFN-beta signaling, the mechanism of dsRNA generation through convergent transcription warrants further investigations in the context of radio/chemotherapy.

Our data indicate that IR and chemotherapy lead to transcriptional up-regulation of certain small non-coding RNAs and their nuclear to cytoplasmic translocation (see Figure 5D-5E and Supplementary Figure S5C-S5D), thus allowing them to bind to RIG-I. Interestingly, RIG-I is a radioinducible protein (Supplementary Figure S6). RT, increases the concentration of active cytoplasmic complexes between these RNA receptors and their ligand RNAs, thereby activating downstream signaling and IFN-beta production. We described this mechanism for U1 and U2, but further comprehensive RNA sequencing experiments are necessary to evaluate the pattern of different cellular RNAs interacting with individual members of RLR pathway in the context of radio- and chemotherapy and to estimate the role of transcriptional and post-transcriptional events in activation of this pathway. The importance of comprehensive characterization of such activating RNAs is emphasized by recent data regarding differential expression in cancer cells of non-coding RNAs with motifs specific for PRRs [51]. Potential immuno-stimulating properties of such activating RNAs and understanding of their “activating” modifications may improve current approaches to the design of RNA-based vaccines [52].

Data described in the current report describe cell-intrinsic RNA responses to DNA damaging agents in tumor and normal cells. However, current literature indicate that RNA signaling can activate pattern recognition receptors using cell extrinsic, paracrine signaling. At least two pathways are described for such extrinsic signaling. One was demonstrated for U1 snRNA, which upon UV damage can leak in the extracellular space and bind to TLR3 receptors [38]. Interestingly, the regions of U1 that were reported sufficient for binding with TLR3 overlap with the stem loop regions we identified to be involved in interactions with RIG-I and subsequent induction of the IFN response (see Figure 5G and 5H and [38]). Such ‘passive” leakage of dsRNAs from irradiated cells can be also essential for TLR3-dependent gastrointestinal injury, recently described by Takemura *et al*. [44]. Another extrinsic RNA-dependent pathway, described by Boelens *et al*., occurs through exosomes, which are secreted by stromal cells in a RAB27B-dependent manner [21]. These exosomes deliver various types of non-coding RNAs to tumor cells, resulting in the activation of the RIG-I/MAVS pathway, which eventually induce the IRDS signature in tumor cells. Interestingly, these exosomes were found to contain non-coding snRNAs and are enriched in Alu/SINE and LINE elements as well as microsatellite RNA [35, 36, 50].

Our data support the importance of RNA-dependent RLR-mediated IFN response to radio/chemotherapy of tumors. However, recently it was demonstrated that another cytoplasmic innate immune pathway – DNA-dependent STING pathway [53] - is also implicated in the tumor response to IR [54]. An intriguing difference is that the requirement for the STING pathway was demonstrated for host immune cells, primarily in myeloid and dendritic cells (DCs), through activation by a cell-extrinsic mode by DNA molecules that are presumably released from irradiated tumor cells through a yet unidentified mechanism. Perhaps, the tumor and host immune cells may have alternative usage of RNA- and DNA-dependent pathways of response to genotoxic stress. Recent findings indicate that the STING pathway may be deficient in certain types of tumor cells [55], which is consistent with sufficiency of RNA-dependent RLR responses to radio/chemotherapy in tumor cells and/or cells of mesenchymal origin, described in this report.

In conclusion, our data provide the first comprehensive demonstration of the role of RIG-I/MAVS pathway in Type I IFN induction in tumor cells exposed to IR and chemotherapy. Our study highlights the unusual role of small endogenous dsRNAs in DNA-damage response (DDR), previously associated almost exclusively with DNA repair/recombination machinery [56]. Targeting the LGP2/RIG-I/MAVS/IFN-beta pathway may provide new strategies for radioprotection after exposure to total body or abdominal irradiation, as well as tumor sensitization to IR. We have also demonstrated that detection of structural elements of RNAs, which bind to RIG-I can be used for optimization of ligands with maximal capacity to induce type I IFNs and therefore activate adaptive immune response (see Figure 5G and 5H). Finally, these data suggest a co-evolution of cellular defenses against pathogens and the response to IR, which warrants further investigations of RLR functions in tumor and normal cells.

## MATERIALS AND METHODS

### Animals

All mice were handled in accordance with animal experimental guidelines approved by the Institutional Animal Care and Use Committee of the University of Chicago. Mice were maintained under specific pathogen-free conditions in a barrier facility. Age-matched 9-12 week old C57BL/6 mice were purchased either from Jackson Lab or Harlan. LGP2^−/−^ and MDA5^−/−^ mice were generous gifts from Dr. Michael Gale, Jr. of the Department of Immunology at the University of Washington. RIG-I^+/−^ in ICR background [58] were obtained from Dr. Balaji Manicassamy of the Department of Microbiology at the University of Chicago, with permission from Dr. Shizuo Akira.

### Cell lines

Primary mouse embryonic fibroblasts (MEFs) from MAVS^−/−^ mice were kindly provided by Dr. Tatyana V. Golovkina of the Department of Microbiology at the University of Chicago. Primary MEFs (wild-type, LGP2^−/−^, RIG-I^−/−^, and MDA5^−/−^) were derived from 13.5 day post-coitus embryos and cultured in DMEM supplemented with 10% FBS, 1% non-essential amino acids, and 1% penicillin/streptomycin (P/S) for no more than five passages. Human embryonic kidney 293 (HEK293) cell lines were utilized for transient transfection experiments. HCT116 (human colon adenocarcinoma), WiDr (human colon adenocarcinoma), and D54 (human glioblastoma multiforme) tumor cell lines were used for knockdown experiments. HEK293 and HCT116 cell lines were grown in DMEM high glucose with 10% FBS and 1% P/S. WiDr and D54 cell lines were cultivated in MEM with 10% FBS and 1% P/S.

### Ionizing radiation exposure

Mice were exposed to 5.5 Gy total body irradiation using the RadSource Technologies X-ray RS-2000 Biological Irradiator operating at 160 kVp and 25 mA at a dose rate of 2.20 Gy/min. Mice were fed Uniprim for the entire duration of the experiment. Mice were carefully monitored every 2-3 days, and blood was collected from the periorbital sinus of anesthetized mice at the indicated time points. The severity of gastrointestinal syndrome was evaluated by observing body mass loss and performing histological analysis of small intestinal tissue sections. Mice that appeared lethargic and moribund were immediately sacrificed. Apoptotic small intestinal cell death was evaluated by TUNEL staining. Human tumor xenografts were established in the right flank of athymic nude mice. Tumors that reached a volume of ~150mm^3^ were locally irradiated at fractionated doses of 5 Gy for a total of 6 consecutive days using the RadSource Technologies X-ray RS-2000 Biological Irradiator. Cell lines maintained in tissue culture were irradiated using a Gammacell 220 (MDS Nordion, Ottawa, Canada) ^137^Cs γ-irradiator.

### Microarray analysis

WT and MAVS^−/−^ primary MEFs were seeded in 6-well plates at a density of 2×10^5^ cells/well. Approximately 15 hours post-seeding, cells were either mock-irradiated (un-irradiated) or exposed to 6 Gy IR. Total RNA was isolated using Trizol reagent following the manufacturer's protocol. RNA yield was measured using Qubit RNA broad range kit. 100 ng of RNA was labeled per the manufacturer's instructions and profiled in duplicate using the Illumina Mice WG-6 array (Illumina, San Diego CA). Background subtraction and quantile normalization was performed across arrays using Illumina Beadstudio software. Log-transformed gene expression was compared using Significance Analysis of Microarrays (SAM) for Excel (Stanford University, CA) with a False Discovery Rate (FDR) of 5% and a fold-change threshold of greater than or equal to 1.5 to identify differentially expressed genes [59]. Ingenuity Pathway Analysis (IPA, Redwood City CA) was used to identify top ranked canonical pathways and cellular functions (*P* < 0.05).

### Quantification of IFN-beta production

Primary MEFs and human tumor cell lines were seeded in 96-well plates at a density of 15,000 cells/well. Approximately 15 hours post-seeding, cells were exposed to IR. Cell culture supernatant were harvested 48 hours post-IR and assayed for IFN-beta using the *VeriKine-HS Mouse Interferon Beta Serum ELISA Kit (PBL Assay Science) and the* Human IFN-beta ELISA kit for murine and human cell lines following the manufacturer's protocol. Absorbance at 450 nm was measured using a BioTek Synergy HT plate reader. IFN-beta concentration was calculated by using standard concentration and fitting a five-parameter logistic non-linear regression model available at the free analysis software ELISA Analysis (http://www.elisaanalysis.com/).

### Quantification of caspase 3/7 activity

Primary MEFs and human tumor cell lines were seeded in 96-well plates at a density of 15,000 cells/well. Approximately 15 hours post-seeding, cells were exposed to IR. 48 hours post-IR treatment, cell culture supernatant were aspirated and replaced with fresh media. Caspase 3/7 reagent was added to each well, mixed at room temperature with shaking at 400 rpm for 5 minutes, followed by incubation in the dark without shaking for 30 minutes. The luminescence of each sample was measured using the BioTek Synergy HT plate reader.

### Clonogenic and cell viability assays

Clonogenic and cell viability assays were performed as described in Widau *et al.*, 2014. Briefly, cells were seeded in corresponding plates at specific densities. Approximately 15 hours post-seeding, cells were exposed to increasing doses of IR. For clonogenic assays, cells were grown until sufficiently large colonies with at least 50 cells were visible (~12 days after IR). Cells were washed in 0.85% NaCl and simultaneously fixed and stained in a solution containing methanol and crystal violet. Colonies with at least 50 cells were counted and the surviving fraction was calculated. For viability assay, cells were stained with 0.4% methylene blue in 50% methanol at 96 hours post-IR treatment. Dye was extracted from stained cells using 3% solution, and absorbance at 660nm was quantified using the BioTek Synergy HT plate reader. In some experiments, the Trevigen XTT Cell Proliferation assay kit was used to assess cell viability.

### HEK293 reconstitution experiments

HEK293 cells were seeded in a 24-well plate overnight at a density of 1.5×10^5^ cells/ml (75,000 cells/well). HEK293 cells were co-transfected with increasing amounts of pEF-BOS-hMAVS, pEF-BOS-RIG-I, or pEF-BOS-MDA5 (Addgene; Cambridge, MA) together with a firefly luciferase gene driven by the IFN-beta promoter and a Renilla luciferase gene driven by a basal promoter (pRL-null) as a transfection control. Transfections were performed using a cationic lipid agent, Fugene-HD (Promega), at a 3:1 lipid:DNA ratio. The total amount of transfected plasmid DNA was equalized by supplementing with empty pEF-BOS vector. Twenty-four hours post-transfection, cells were either mock-irradiated or exposed to 6 Gy IR. Forty-eight hours post-IR treatment, cell lysates were collected and 20 μl were transferred to opaque 96-well plates. Following the manufacturer's protocol for the dual luciferase assay (Promega), samples were analyzed for IFN-beta luciferase and Renilla luciferase activity using a BioTek Synergy HT plate reader (BioTek; Winooski, VT). The transfection efficiency across different wells was normalized by dividing the firefly luciferase activity by the Renilla luciferase control. After correcting for transfection efficiency, all values were normalized to those of non-irradiated cells transfected with the empty pEF-BOS vector.

### Reconstitution experiments in MAVS^−/−^ and RIG-I^−/−^ primary MEFs

MAVS^−/−^ and RIG^−/−^ primary MEFs were transfected with the pEF-BOS-hMAVS and pEF-BOS-hRIG-I constructs following the Amaxa Nucleofector protocol for primary MEFs. Briefly, 2×10^6^ cells were resuspended in 100 μl room-temperature MEF Nucleofector solution 2 and mixed with 5 μg plasmid DNA construct. The cell/DNA suspension was transferred to a cuvette and electroporated in a Nucleofactor 2 machine using the A-023 pre-set program for MEFs. Twenty-four hours post-transfection, cells were trypsinized, counted, and re-seeded for additional assays such as IFN-beta ELISA, caspase 3/7 activity, and cell viability assays.

### siRNA-mediated knockdown of MAVS, RIG-I, and MDA5

Human tumor cell lines were transiently transfected with specific siRNA constructs to knockdown MAVS and RIG-I (Dharmacon). Non-targeting siRNA was used as a control. Twenty-four hours post-transfection, cells were either mock-irradiated or exposed to increasing doses of IR. Cells were then assayed for IFN-beta production, apoptotic activity, and viability.

The following table summarizes the siRNA constructs used in this study and the concentration used for transfection experiments:

**Table:** 

Designation	Name	Dharmacon Catalog No.	Transfection concentration
Scrambled	siGENOME Non-Targeting siRNA	D-001210-02-05	D54: 50 nM
siMAVS	siGENOME Human MAVS siRNA	D-024237-02-0020	WiDr: 30 nM
siRIG-I#1	siGENOME Human DDX58 siRNA	D-012511-01-0005	HCT116: 25 nM
siRIG-I#2	siGENOME Human DDX58 siRNA	D-012511-03-0005	HEK293: 25 nM

### Stable shRNA-mediated MAVS and RIG-I knockdown

Tumor cell lines were transfected with shMAVS construct within a TRC2-pLKO-puro vector backbone (Sigma-Aldrich mission shRNA #236030) using Fugene HD transfection reagent at 1:3 plasmid DNA:lipid ratio. The TRC2 pLKO.5-puro non-targeting shRNA for vertebrates (Sigma-Aldrich SHC216) was used as a control. Stable lines were selected by growth in culture media containing 5ug/ml Puromycin over multiple passages. Successful knockdown of MAVS was confirmed by Western blot. Stable cell lines were assessed for IFN-beta production, caspase 3/7 activity, and clonogenic survival.

Tumor cell lines were transfected with shRIG-I construct within a psiRNA-h7SK GFPzeo backbone (InvivoGen) using Fugene HD transfection reagent at 1:2 plasmid DNA:lipid ratio. psiRNA-LucGL2 in the same backbone served as a non-targeting control. Stable lines were selected by growth in culture media containing 0.25 mg/ml Zeocin over multiple passages. Successful knockdown of RIG-I was confirmed by Western blot. Stable cell lines were assessed for IFN-beta production and caspase 3/7 activity and used for *in vivo* animal studies.

### *In vivo* tumor model in athymic nude mice

100ul of 1 × 10^6^ cultured D54 tumor cells with stable RIG-I knockdown (or a non-targeting control) were injected subcutaneously in the hind limb of athymic nude mice. Tumor volumes were measured along 3 orthogonal axes (l, w, and h) and calculated using the formula for an ellipsoid (l x w x h/2). Tumors were locally irradiated upon reaching a volume of 150mm^3^ as described above. Tumor growth was monitored every 3-4 days, and the relative increase in tumor volume was reported as the ratio of the tumor volume relative to its original tumor volume at the start of IR treatment (V/V_o_). Mice were sacrificed when tumor sizes reached a volume of 2000-3000 mm^3^.

### RNA purification from RIG-I complexes

HEK293 cells were transiently transfected with 3xFLAG-tagged full-size RIG-I in pEF-BOS vector (Addgene; Cambridge, MA). Twenty-four hours post-transfection, cells were either mock-irradiated or exposed to IR (6 Gy). Forty-eight hours post-IR, cells were lysed with a modified lysis buffer (50 mM Tris-Cl pH 7.5, 0.15 M NaCl, 0.1% NP-40, 1.0% Triton X-100, 1 mM EDTA pH8.0, 1 mM EGTA pH8.0, 10% Glycerol, 2.5 mM MgCl_2_, 1 mM DTT, 0.1 mM ATP, and 1X Halt Protease Inhibitor) and incubated on ice for 1 hour. Cell lysates were separated from cell debris by centrifugation at 12,000 rpm at 4°C. Protein concentration was measured by BCA kit. Anti-FLAG monoclonal antibody was added to the cell lysate at a 1:500 dilution, and incubated overnight at 4°C. Protein G sepharose beads were added to the lysates and incubated for at least 2 hours at 4°C. Beads containing the antibody-RIG-I complexes were washed five times in wash buffer (50 mM Tris-Cl pH 7.5, 0.15 M NaCl, 1 mM MgCl_2_, 0.05% NP-40, 1 mM DTT) and proteins were eluted from the beads using a soft elution buffer (0.5% SDS, 0.1% Tween-20, 50 mM Tris pH 8.0) for 10 minutes at room temperature with vortexing every 2-3 minutes. Proteinase K was added to the eluates and incubated at 50°C for 45 minutes. Trizol reagent was then added to the solution, and RNA bound to RIG-I was purified following manufacturer's protocol. RNA quality was analyzed using an Agilent Bioanalyzer 2100 with Pico Series II cartridges. RNA yield was measured using the Qubit RNA Broad Range kit. For qRT-PCR validation experiments of RIG-I pulled down RNA, UV cross-linking (2 doses at 150mJ/cm^2^) was performed on HEK293 and HCT116 overexpressing RIG-I prior to cell lysis. The same protocol for pulldown and RNA purification experiments was performed as described above.

### RNA purification from cytoplasmic and nuclear fraction of cells

HEK293 and HCT116 cells were seeded in 6-well plates overnight at a density of 2 × 10^5^ cells/ml. Cells were irradiated at 6 Gy and harvested at 6, 24, 48 and 72 hours post-IR treatment. The cytosolic and nuclear fractions were prepared following the protocol reported by Liu and Fagotto [60]. Briefly, cells were washed with PBS then incubated with cold digitonin solution (42ug/ml digitonin, 2mM DTT, and 2mM MgCl_2_ dissolved in 1x NEH buffer – 150mM NaCl, 0.2mM EDTA, 20mM Hepes-NaOHat pH 7.4) for 15 minutes at 4°C in an orbital shaker. The digitonin-solubilized material, which contains the cytosolic fraction, was transferred to 1.5ml tubes. To the remaining cell debris on the plate (the nuclear and mitochondrial fraction), cold PBS was added to remove traces of the digitonin solution and was subsequently lysed with lysis buffer (50mM Tris-Cl pH 7.5, 0.1% NP-40, 1% Triton X-100, 10% glycerol, 0.15M NaCl, 1mM EDTA, 1mM EGTA, and 1% SDS). The lysates were transferred to 1.5ml tubes. To purify RNA from both cytosolic and nuclear fractions, equal volume of acid phenol was added to the samples. The aqueous portion was precipitated with equal volume of isopropanol and 1ul glycogen. The cell pellet was washed with 75% ethanol and dissolved in RNase-free water.

### RNA stimulation of HEK293 IFN-beta reporter cells

HEK293 cells were seeded in a 24-well plate overnight at a density of 1.5 × 10^5^ cells/ml (75,000 cells/well). Cells were co-transfected with 100 ng of plasmid construct (pCAGGS empty vector, pCAGGS-RIG-I full-length, pCAGGS-RIG-I helicase-RD mutant construct, and pCAGGS-RIG-I K858A-K861A mutant construct), together with 80 ng of a firefly luciferase reporter gene driven by an IFN-beta promoter and 20 ng of a Renilla luciferase (pRL-null) transfection control. Transfections were performed using a cationic lipid agent, Fugene HD (Promega), at a 3:1 lipid:DNA ratio. Twenty-four hours post-transfection, cells were then stimulated with 1 μg RNA* mixed with Fugene HD at 2:1 lipid: RNA ratio for 24 hours. 20 μl cell lysates were collected in opaque 96-well plates and analyzed for IFN-beta-luciferase and Renilla activity using a BioTek Synergy HT plate reader. The transfection efficiency across different wells was normalized by dividing the IFN-beta luciferase activity with the Renilla activity. All values were further normalized to the unstimulated controls in cells transfected with the empty vector. All pCAGGS RIG-I constructs used in this study were generous gifts from Dr. Jenish Patel and Dr. Adolfo Garcia-Sastre of The Icahn School of Medicine at Mount Sinai in New York City.

*Total RNA stimulation: Total RNA from donor HEK293 cells was prepared from irradiated cells and harvested at different time points post-IR treatment (24, 48 and 72 hours post-IR). Trizol reagent was used to purify the total RNA. RNA yield was measured using Qubit RNA broad range kit.

*Synthetic RNA stimulation: Synthethic RNA comprised of various stem loop regions of the human U1 snRNA were purchased from IDT Oligos as reported in [38]. U1 stem loop I sequence: 5′-GGGAGAACCAUGAUCACGAAGGUGGUUUUCCC-3′; U1 stem loop II sequence: 5′-GGGCGAGGCUUAUCCAUUGCACUCCGGAUGUGCUCCCC-3′; U1 stem loop III sequence: 5′-CGAUUUCCCCAAAUGUGGGAAACUCG-3′; U1 stem loop IV sequence: 5′-UAGUGGGGGACUGCGUUCGCGCUUUCCCCUG-3′; U1 stem loops I and II sequence: 5′-GGGAGATACCATGATCACGAAGGTGGTTTTCCCAGGGCGAGGCTTATCCATTGCACTCCGGATGTGCTGACCCC-3′; U1 stem loops II and III sequence: 5′-GGGCGAGGCTTATCCATTGCACTCCGGATGTGCTGACCCCTGCGATTTCCCCAAATGTGGGAAACTCGACTGC-3′.

*Double-stranded positive and negative RNA controls were purchased from InvivoGen (San Diego, CA).

Positive control (19-mer):

5′-pppGCAUGCGACCUCUGUUUGA-3′
3′-CGUACGCUGGAGACAAACU-5′;

Negative control (19-mer):

5′-GCAUGCGACCUCUGUUUGA-3′
3′-CGUACGCUGGAGACAAACU-5′

*In vitro transcribed U1 RNA stimulation: Full length U1 (pT7U1) plasmid was generously provided by Dr. Joan Steitz (Yale School of Medicine, Yale University). *In vitro* transcription was performed using the HiScribe T7 Quick high yield RNA synthesis kit (New England Biolabs) following manufacturer's protocol. RNA was purified using the Trizol method.

### RNA sequencing analyses

We eluted total RNA and RNA bound to RIG-I from RIG-I over-expressing HEK293 cells 48 hours post IR (6 Gy). RNA purified from RIG-I pulldown as well as total RNA from HEK293 cells were used as templates to generate cDNA libraries for RNA sequencing using strand-specific NEBNext Ultra RNA Library Prep Kit for Illumina (New England Biolabs) following RiboZero (Epicentre) treatment for rRNA depletion. Libraries were sequenced on Illumina HiSeq2500 instrument to generate 50 bp pair-ended reads. Sequencing files in FastQ format were processed using AlienTrimmer [61] to remove adapter sequences and to trim low quality reads with Phred quality score < 20. The preprocessed reads were aligned to the human reference genome (Ensembl GRCh38) using Spliced Transcripts Alignment to a Reference (STAR) software [62]. The featureCounts tool from Bioconductor package RSubread was used to summarize and quantify the abundances of genomic features of the mapped reads [63]. Mapped reads were annotated using human GENCODE version 20 [64] and were summarized to 35 gene/transcript and non-coding RNA biotypes annotated in GENCODE/Ensemble databases. RepEnrich program [65] was used to identify and quantify the repetitive elements. The program uses Bowtie [66] to align the reads to the human reference genome (Ensembl GRCh38) and human repetitive element pseudogenomes built upon RepeatMasker annotation library hg38.fa.out.gz (http://www.repeatmasker.org). The mapped reads were summarized by repetitive element subfamilies, families and classes (Supplementary Data Table 1). To identify differentially expressed genomic features among RIG-I pulldown samples and total RNA (±IR treatment) samples, Bioconductor package DESeq2 [67] and limma [68, 69] were used.

### Western blotting antibodies

For confirmation of targeted knockdown experiments as well as transient transfection/reconstitution experiments in both murine and human cell lines, the following primary antibodies were used: anti-hMAVS (sc-166583; Santa Cruz Biotechnology), anti-mMAVS (#4983; Cell Signaling Technology), anti-hRIG-I (70R-16795; Fitzgerald Industries International), anti-mRIG-I (#3743; Cell Signaling Technology), anti-MDA5 (#5321; Cell Signaling Technology), anti-LGP2 (70R-16832; Fitzgerald Industries International), anti-TBK1 (sc-9910; Santa Cruz Biotechnology), anti-phospho-TBK1 (S172 clone D52C2; 5483S; Cell Signaling Technology), anti-IRF-3 (clone FL-425; sc-9082; Santa Cruz Biotechnology), anti-FLAG (M2 clone; Sigma), anti-HA (Y-11 clone; sc-805; Santa Cruz Biotechnology), anti-α-Tubulin (sc-12462R; Santa Cruz Biotechnology), and anti-Actin-HRP (sc-47778; Santa Cruz Biotechnology). Secondary antibodies conjugated to HRP (Santa Cruz Biotechnology) were used at a 1:10,000 dilution.

### qRT-PCR analysis

1 μg total RNA was subjected to DNase I treatment in a 30 μL reaction volume using DNase I, RNase-free (Thermo Scientific) following the manufacturer's protocol. cDNA was synthesized from 10 μL of the DNase treated RNA using the High-Capacity cDNA Reverse Transcription Kit (LifeTechnologies) following the manufacturer's protocol. A mock-RT (no reverse transcriptase) control reaction was also performed. cDNAs were diluted 1:10 with 0.1X TE, and stored at -20°C until use.

96-well plate-based triplicate qPCR reactions were assembled in a 15 μL final volume using 2X Power SYBR^®^ Green Master Mix (LifeTechnologies) with 1/10 volume of 5 μM each PCR primer. Thermal cycling, data collection and dissociation curve generation were performed on a BioRad iCycler IQ5. Mock irradiated control reactions either did not generate a signal or were approximately 10 units greater than the plus RT reactions.

The following primers were used for validation of the microarray data:

**Table:** 

Target Gene	Forward primer sequence	Reverse primer sequence
*Usp18*	ACAGCCCTCATGGTCTGGTTGGT	CTCTCTTCTGCACTCCGAGGCACT
*Ifit3*	AGTGAGGTCAACCGGGAATCT	TCTAGGTGCTTTATGTAGGCCA
*Stat1*	AGTCGGAGGCCCTAATGCT	CCATAATGCACCCATCATTCCA
*Cdkn1a*	CGGTGTCAGAGTCTAGGGGA	CGAAGTCAAAGTTCCACCGT
*Ddx58*	AGAGCCAGCGGAGATAACAA	CCTTGATCATGTTCGCCTT
*Gapdh*	AACGACCCCTTCATTGAC	TCCACGACATACTCAGCAC
*Tbp*	GGTTTCTGCGGTCGCGTCATT	GGTGGAAGGCTGTTGTTCTGGTCC
*B2m*	CTGACCGGCCTGTATGCTAT	CGGGTGGAACTGTGTTACG

Fold change calculations were determined using the 2^-ddCt method normalizing to the geometric mean of *Gapdh*, *B2m* and *Tbp*.

For the quantification of U1 and U2 RNA in RNA purified from pulldown experiments and in fractionated nuclear and cytosolic portions of cell lines, qRT-PCR was conducted on cDNA prepared from DNase I-treated RNA samples, and the following primers were used:

**Table:** 

Target gene	Forward primer sequence	Reverse primer sequence
*U1*	GGAGATACCATGATCACGAAGG	CCACAAATTATGCAGTCGAGTTT
*U2*	AGTTTAATATCTGATACGTCCTCTATCC	GGTCGATGCGTGGAGTG
*18S*	GTAACCCGTTGAACCCCATT	CCATCCAATCGGTAGTAGCG
*GAPDH*	TGCACCACCAACTGCTTAGC	GGCATGGACTGTGGTCATGAG
*B-ACTIN*	TGACATTAAGGAGAAGCTGTCCTAC	GAGTTGAAGGTAGTTTCGTGGATG

Reactions were run on the BioRad iCycler IQ5 in a final volume of 25ul with 4.0uM of the U1 (or U2) forward and reverse primers (or 0.8uM of the forward and reverse primers for *18S*, *GAPDH*, and *β-actin*), using 2x Power SYBR^®^ Green Master Mix (LifeTechnologies). Cycling conditions were a single denaturing step at 95°C for 10mins, followed by 40 cycles of 95°C for 15 seconds and 60°C for 1 minute. Fold change calculations were determined using the 2^-ddCt method normalizing the U1 or U2 values to the geometric mean of *18S, GAPDH*, and β*-ACTIN*. Fold change in irradiated samples was then normalized to non-irradiated controls for the pulldown experiments, while the fold change in the cytoplasmic fraction was normalized to that of the nuclear fraction at each time point post-IR treatment.

### Statistical analysis of human cancer data sets

Clinical cancer data sets and corresponding microarray gene expression data were downloaded from Gene Expression Omnibus (GEO). Matched pre- and post-treatment expression data were obtained using the following accession files: GSE3578 – cervical cancer (radiation n=20 and chemoradiation n=19 paired samples); GSE48277 – bladder cancer (chemotherapy n=20 paired samples); GSE18728 – breast cancer (chemotherapy n=16 paired samples at the time of surgery); GSE21974 – breast cancer (chemotherapy n=25 paired samples). List of Type I ISGs was obtained from [39]. Pre-operative chemotherapy breast cancer datasets used to examine the association of Type I ISG expression, complete pathologic responses (pCR), and distant relapse-free survival following doxorubicin-based neoadjuvant chemotherapy were downloaded using the following accession numbers: GSE25055 (n=310), GSE25065 (n=198) and GSE20194 (n=278). Probe intensities were quantified with MAS 5.0 using Affymetrix default analysis settings. CEL files were normalized using global scaling with a trimmed mean target intensity of each array arbitrarily set to 600 using the MAS5 algorithm from the simpleaffy package. Normalized expression values were renormalized to the median value across all patients within each respective data set. Complete pathologic responses were available for patients within each data set. All statistical analyses were performed using JMP 9.0 (SAS Institute).

## SUPPLEMENTARY FIGURES AND TABLE




