# Supplementary material for: Cancer therapies activate RIG-I-like receptor pathway through endogenous non-coding RNAs
Source: Oncotarget. 2016 Mar 28;7(18):26496–515. doi: 10.18632/oncotarget.8420 (PMC5041995; doi:10.18632/oncotarget.8420)
Supplement: Supplementary file 1 [file oncotarget-07-26496-s001.pdf]

Cancer therapies activate RIG-I-like receptor pathway through endogenous non-coding RNAs

SUPPLEMENTARY FIGURES AND TABLE

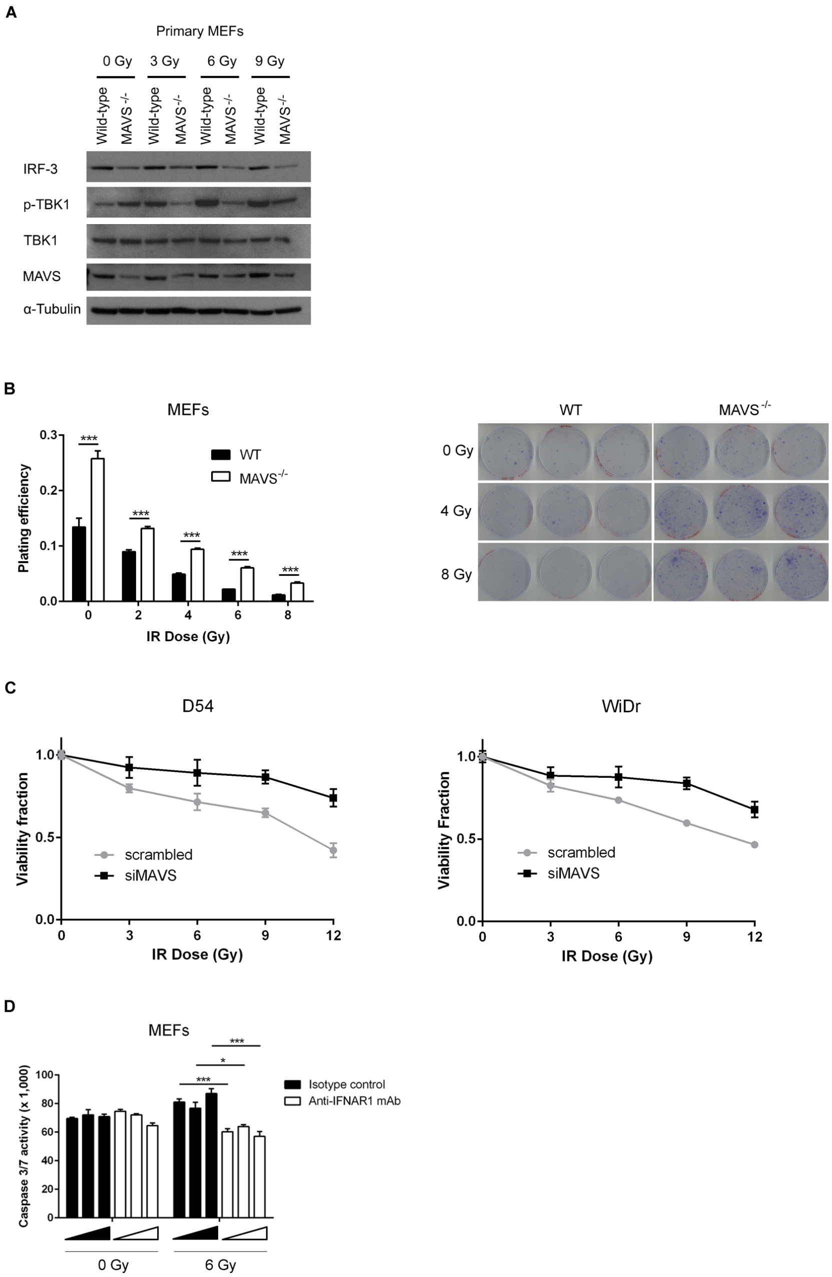

Supplementary Figure S1: MAVS is required for IR-induced cell killing. A. Western blot analyses of lysates from WT and MAVS$^{-/-}$ primary MEFs 48 hours post-exposure to increasing doses of IR. The membranes were probed for MAVS, TBK1, phospho-TBK1, and IRF3. α-Tubulin antibody was used for loading control. **B.** Clonogenic survival of immortalized C57BL/6 wild-type (WT) and MAVS$^{-/-}$ MEFs after exposure to increasing doses of IR (left). Representative scanned images of colonies are shown (right). **C.** Cell viability after siRNA-mediated suppression of MAVS (siMAVS) in the human D54 glioblastoma (left) and WiDr colon adenocarcinoma cell lines (right) in the response to IR as compared to a scrambled transfection controls. **D.** Wild-type primary MEFs were pre-incubated with neutralizing anti-IFNAR1 monoclonal antibody (1, 10, or 50 μg/ml) or an isotype control 90 minutes prior to IR treatment. Apoptotic induction was assessed by measurement of caspase 3/7 activation. $*P < 0.05$, $***P < 0.005$.

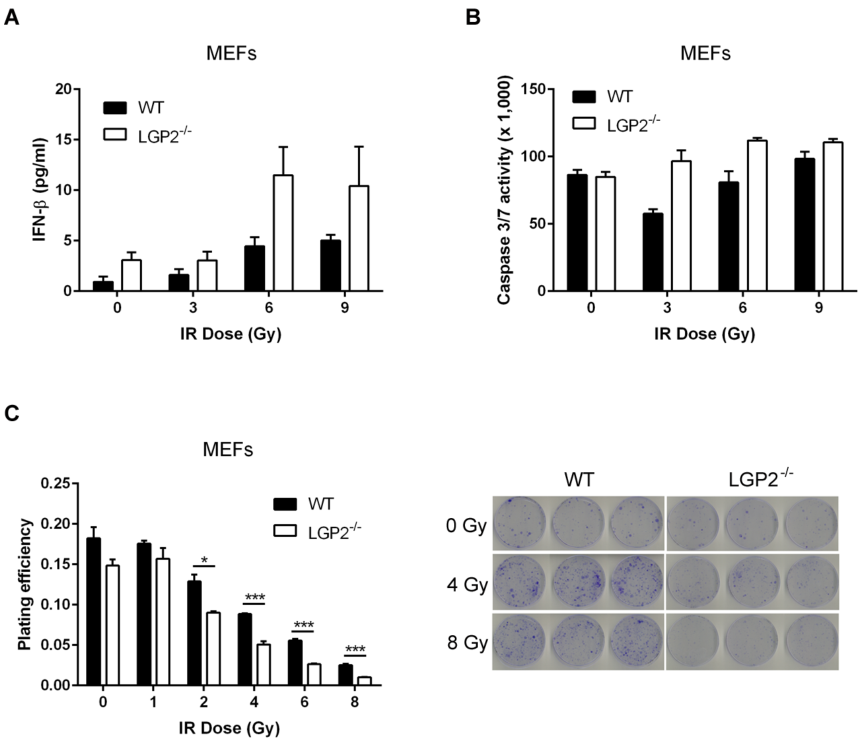

Supplementary Figure S2: LGP2 suppresses IFN-beta-dependent cytotoxicity. Wild-type (WT) and LGP2$^{-/-}$ MEFs were assessed for IFN-beta secretion **A.**, caspase 3/7 activity **B.**, and clonogenic survival **C.** following exposure to increasing doses of IR. Representative scanned images of colonies are shown (right). *$P < 0.05$, ***$P < 0.005$.

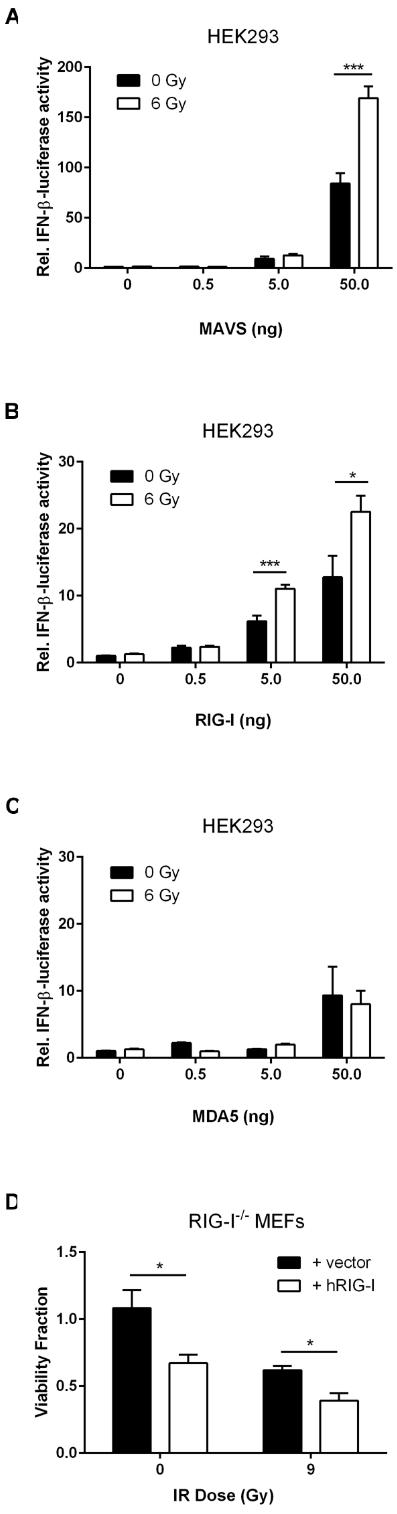

Supplementary Figure S3: MAVS and RIG-I promote IFN-beta expression following IR treatment. Ectopic overexpression of MAVS **A.**, RIG-I **B.**, and MDA5 **C.** inHEK293 cells co-transfected with the IFN-beta promoter-driven luciferase reporter and a Renilla reporter construct. Cells were subsequently irradiated 24 hours following transfection. Luminescence was measured at 48 hours and the relative IFN-beta luciferase activity was normalized to the non-irradiated cell control transfected with the empty vector. **D.** RIG-I mediates cell survival following exposure to IR. Cell viability of RIG-I$^{-/-}$ MEFs reconstituted by full-length human RIG-I or transfected with an empty vector. $*P < 0.05$, $***P < 0.005$.

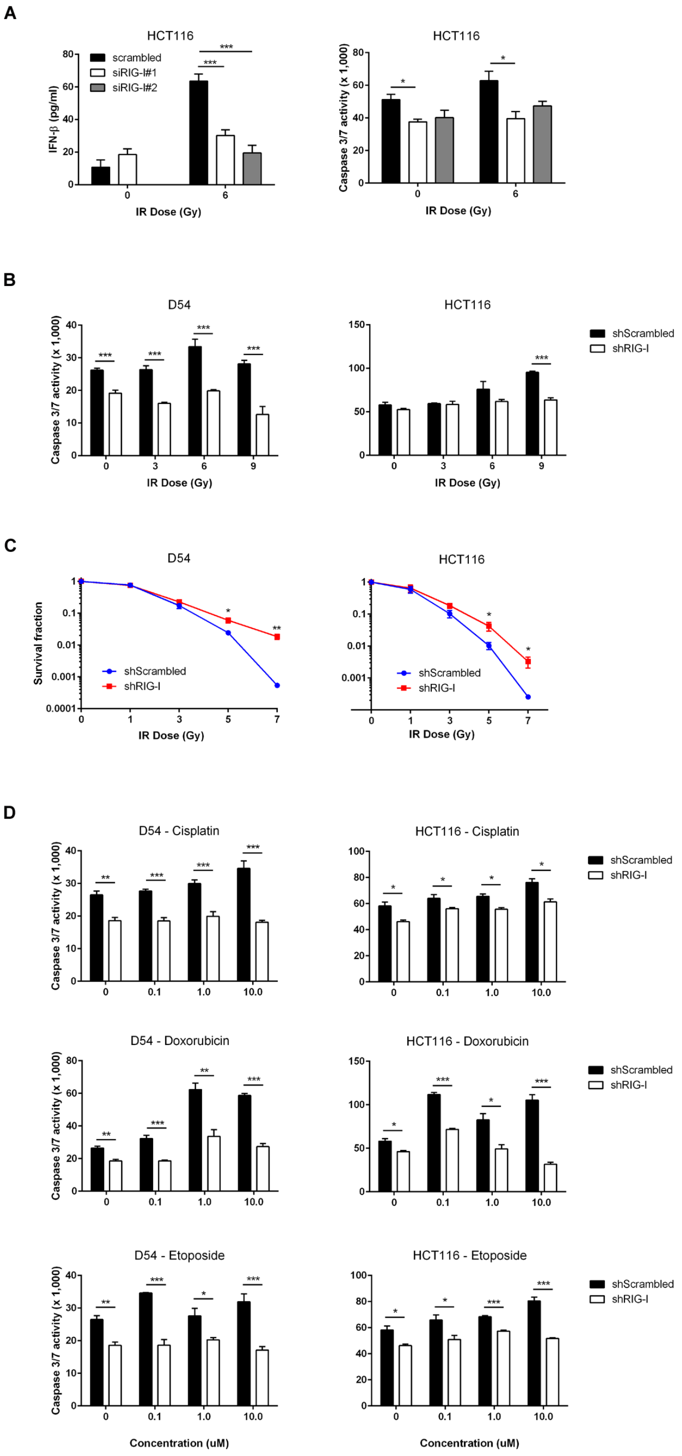

Supplementary Figure S4: RIG-I mediates apoptotic responses to IR and genotoxic chemotherapy drugs. A. IFN-beta protein secretion and caspase 3/7 activation 48 hours post-IR in HCT116 cells treated with siRNA targeting RIG-I. **B.** Caspase 3/7 activities after stable RIG-I knockdown (shRIG-I) of D54 and HCT116 tumor cells. **C.** Clonogenic survival of D54 and HCT116 shRIG-I. Depletion of RIG-I increased clonogenic D_o values from 0.95 ± 0.009 Gy to 1.68 ± 0.15 Gy (p=0.001) in D54 and from 0.86 ± 0.018 Gy to 1.23 ± 0.119 Gy (p=0.006) in HCT116 cells. Anticancer treatment consisted of increasing doses of IR (B), cisplatin, doxorubicin or and etoposide **D.** In all treatments, Caspase 3/7 activation 48 hours post-IR was used as read-out. Control cells were transfected with scrambled shRNA constructs. Scrambled – scrambled siRNA control; si-RIG-I#1 – siRIG-I construct #1; si-RIG-I#2 – siRIG-I construct #2; shScrambled – scrambled shRNA control; shRIG-I – shRIG-I plasmid construct. $*P < 0.05$, $**P < 0.01$, $***P < 0.005$.

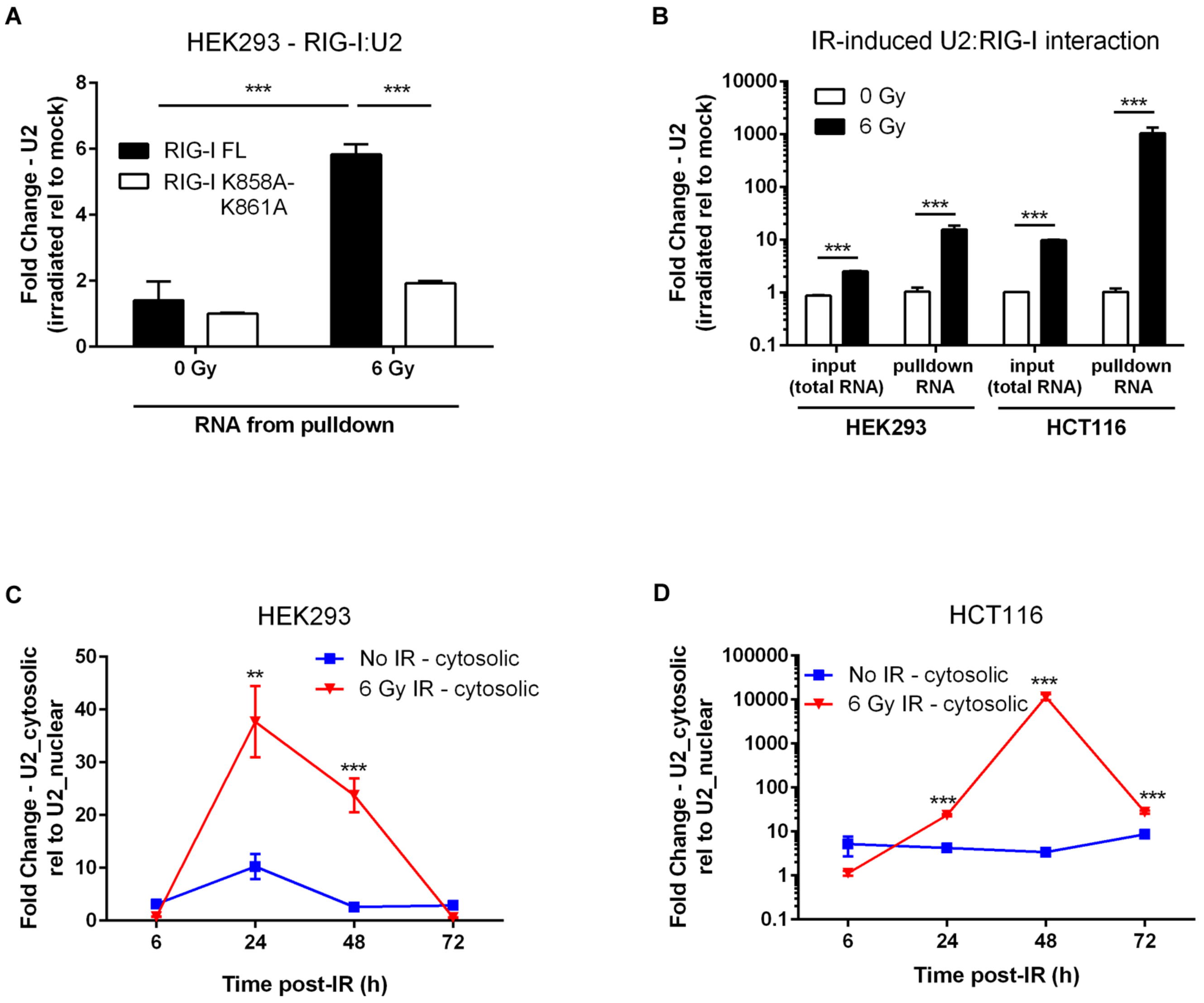

Supplementary Figure S5: U2 is enriched in RIG-I: RNA complexes and redistributes to the cytosol following irradiation. A. Quantification of U2 levelsin RNA purified from RIG-I pulldown in HEK293 cells overexpressing either the full length RIG-I or the K858A-K861A RNA binding deficient mutant. **B.** Quantification of U2 levels in total cellular input RNA and pulldown RNA purified from RIG-I overexpressing HEK293 and HCT116 cells. For both (A) and (B), fold change in irradiated samples was normalized to the un-irradiated controls. The time courses of nuclear and cytoplasmic redistribution of U2 were quantified in both HEK293 **C.** and HCT116 **D.** post-IR. Fold change in the cytoplasmic fraction was normalized to the nuclear levels of U2 for each time point. $*P < 0.05$, $**P < 0.01$, $***P < 0.005$.

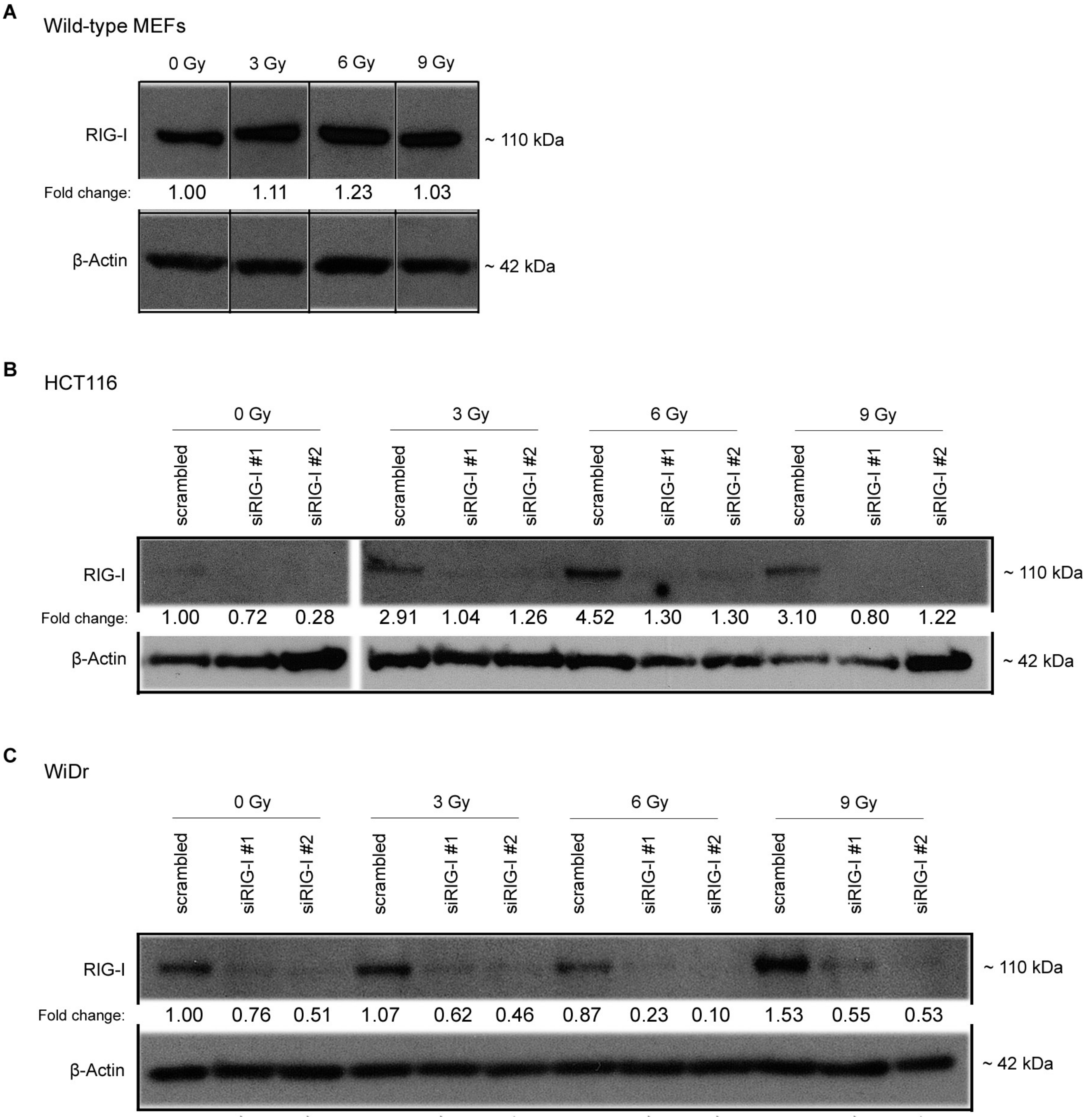

Supplementary Figure S6: RIG-I protein expression is induced by ionizing radiation. Western blot analyses of cell lysates from C57BL/6 wild-type MEFs **A.**, as well as HCT116 **B.** and WiDr tumor cell lines **C.** harvested 48 hours post-IR treatment at increasing doses. For (B) and (C), targeted siRNA was used to knock-down RIG-I in human tumor cell lines. The band intensities were quantified using ImageJ software, and the reported values were normalized relative to the non-irradiated control per cell line. Scrambled - scrambled siRNA control, siRIG-I #1 – siRIG-I construct #1, siRIG-I #2 – siRIG-I construct #2.

A

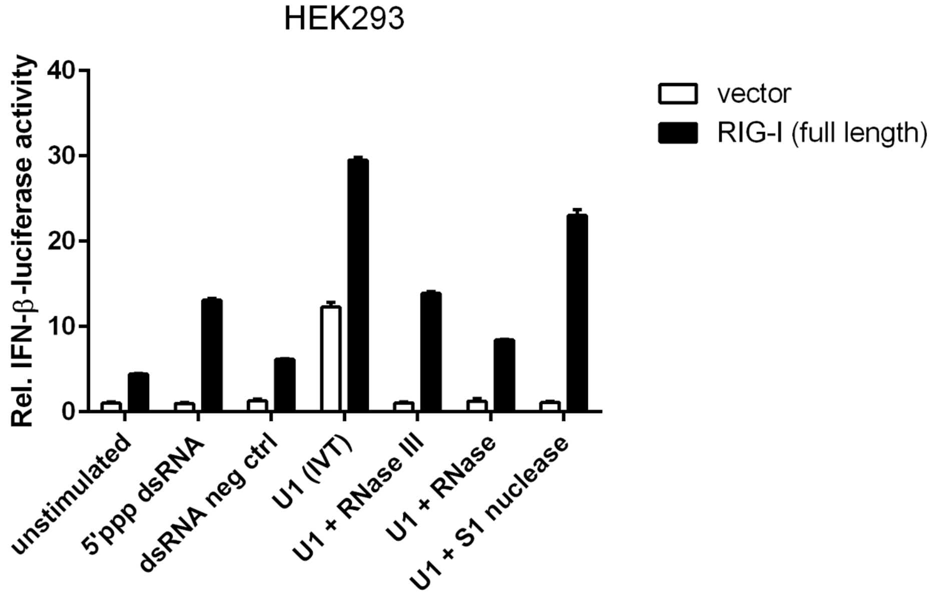

B

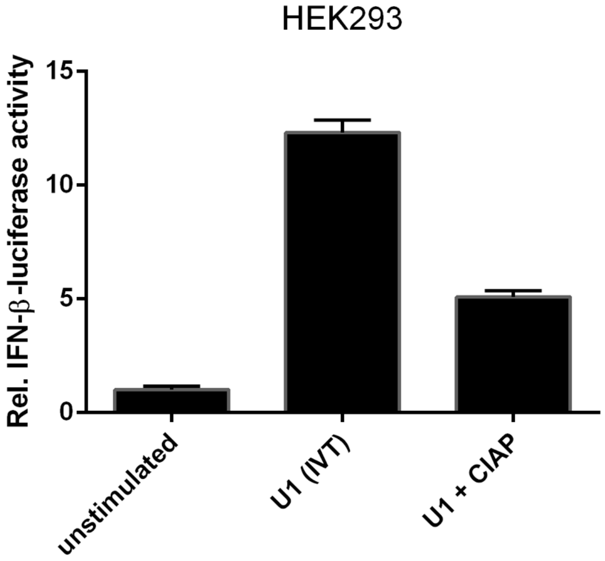

Supplementary Figure S7: Full length *in vitro* transcribed U1 snRNA stimulates endogenous and ectopically expressed RIG-I in HEK293 IFN-beta luciferase reporter cells. A. Relative IFN-beta luciferase reporter activity in HEK293 cells stimulated for 24 hours with *in vitro* transcribed full length U1 snRNA. HEK293 cells were transfected with either an empty vector or the full length RIG-I. In addition, U1 was digested one hour before HEK293 stimulation by treatment with various nucleases: dsRNA-specific RNase III, RNase A, and single-strand specific nuclease S1. The positive and negative controls used in this experiment were the 5'-triphosphorylated 19-mer dsRNA and the corresponding unphosphorylated counterpart, respectively. **B.** CIAP treatment of U1 reduced induction of IFN-beta promoter in HEK293 cells.

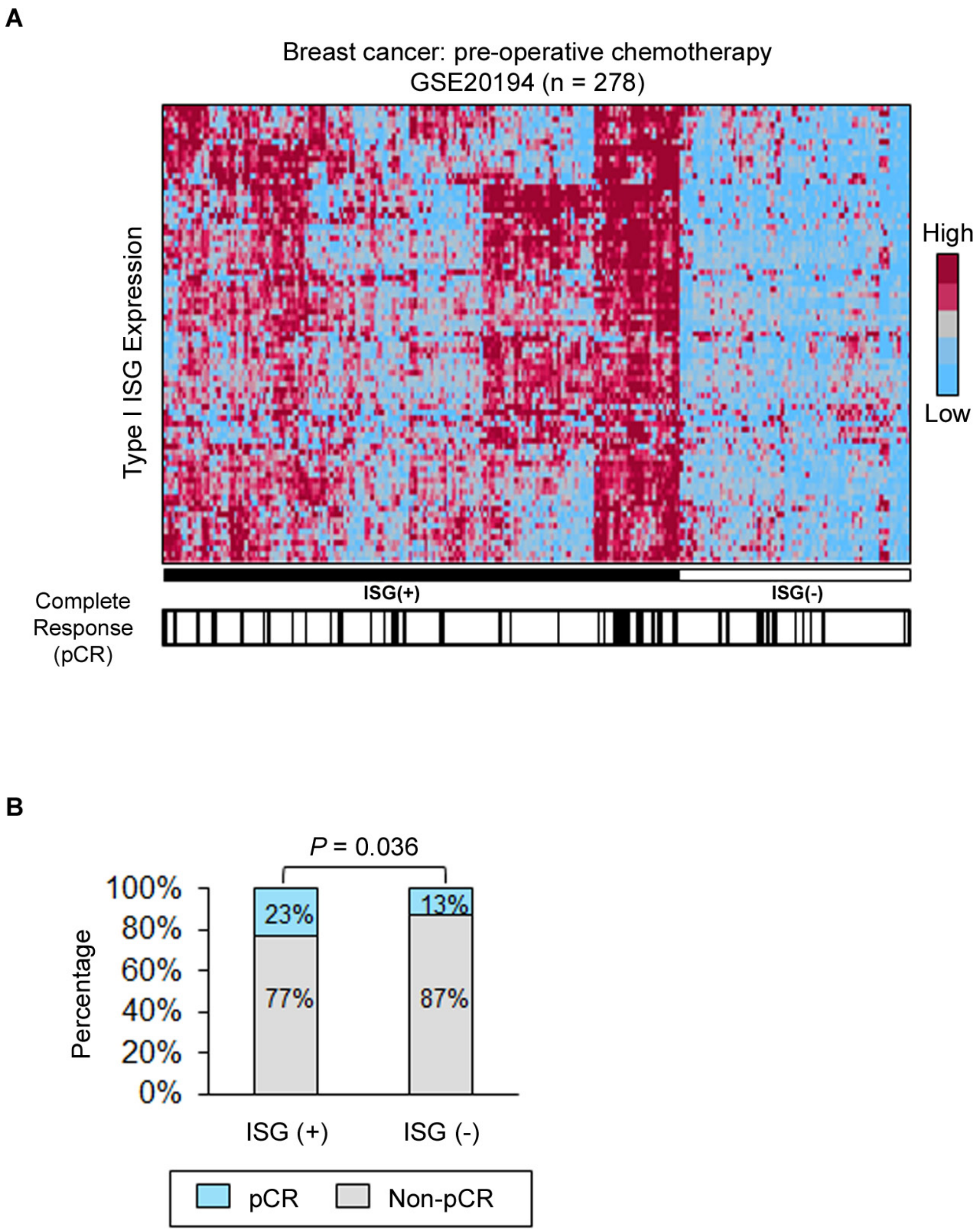

Supplementary Figure S8: Type I interferon-stimulated gene expression is associated with improved responses to pre-operative chemotherapy. A. Heatmap of 81 Type I ISGs distinguishing two molecular subgroups of breast cancer patients (GSE20194, n=278). ISG(+) defined by overexpression of type I ISGs (left). Black hash marks denote complete pathologic response (pCR) to pre-operative doxorubicin-based chemotherapy. **B.** Frequency of pCR in ISG(+) and ISG(-) breast cancer patients treated with pre-operative doxorubicin-based chemotherapy. *P* value was determined by using Fisher's exact test.

Supplementary Table S1: RepeatMasker annotation of RIG-I-bound RNAs

See Supplementary File 1
